# Nickel(II) carbonyl, ammonia, and aceto­nitrile complexes supported by a pyridine dipyrrolide pincer ligand

**DOI:** 10.1107/S2056989020013341

**Published:** 2020-10-09

**Authors:** H. V. Rasika Dias, Abhijit Pramanik

**Affiliations:** a Department of Chemistry and Biochemistry, The University of Texas at Arlington, Arlington, Texas 76019, USA

**Keywords:** crystal structure, nickel, pincer ligand, N-donors, carbon monoxide

## Abstract

The synthesis, isolation and crystal structures of nickel(II) carbonyl, aceto­nitrile and ammonia complexes supported by a dianionic, pyridine dipyrrolide pincer ligand [pyrr_2_py]^2−^, namely, carbonyl[2,2′-(pyridine-2,6-di­yl)bis­(3,5-di-*p*-tolyl­pyrrolido-κ*N*)]­nickel(II), [Ni(C_41_H_33_N_3_)(CO)], ammine[2,2′-(pyridine-2,6-di­yl)bis­(3,5-di-*p*-tolyl­pyrrolido-κ*N*)]nickel(II), [Ni(C_41_H_33_N_3_)(NH_3_)], and (aceto­nitrile-κ*N*)[2,2′-(pyridine-2,6-di­yl)bis­(3,5-di-*p*-tolyl­pyrrolido-κ*N*)]nickel(II), [Ni(C_41_H_33_N_3_)(CH_3_CN)], as well as the free ligand 2,6-bis­(3,5-di-*p*-tolyl­pyrrol-2-yl)pyridine, C_41_H_35_N_3_ or [pyrr_2_py]H_2_ are reported. The nickel complexes are four-coordinate and adopt a square-planar geometry. The CO stretch of the nickel-bound carbon monoxide ligand of [pyrr_2_py]Ni(CO) has been observed at 2101 cm^−1^. The ammonia and aceto­nitrile complexes, [pyrr_2_py]Ni(NH_3_) and [pyrr_2_py]Ni(NCMe) feature all-nitro­gen coordination spheres around nickel consisting of different N-donor ligand types.

## Abstract 2

The synthesis, isolation and crystal structures of nickel(II) carbonyl, aceto­nitrile and ammonia complexes supported by a dianionic, pyridine dipyrrolide pincer ligand [pyrr_2_py]^2−^, namely, carbonyl[2,2′-(pyridine-2,6-di­yl)bis­(3,5-di-*p*-tolyl­pyrrolido-κ*N*)]­nickel(II), [Ni(C_41_H_33_N_3_)(CO)], ammine[2,2′-(pyridine-2,6-di­yl)bis­(3,5-di-*p*-tolyl­pyrrolido-κ*N*)]nickel(II), [Ni(C_41_H_33_N_3_)(NH_3_)], and (aceto­nitrile-κ*N*)[2,2′-(pyridine-2,6-di­yl)bis­(3,5-di-*p*-tolyl­pyrrolido-κ*N*)]nickel(II), [Ni(C_41_H_33_N_3_)(CH_3_CN)], as well as the free ligand 2,6-bis­(3,5-di-*p*-tolyl­pyrrol-2-yl)pyridine, C_41_H_35_N_3_ or [pyrr_2_py]H_2_ are reported.

## Chemical context   

Pincer ligands were first introduced by Moulton and Shaw in 1976 (Moulton & Shaw, 1976 ▸). They are utilized widely as auxiliary ligands to produce transition-metal complexes useful in a range of applications including catalysis (Alig *et al.*, 2019 ▸; Peris & Crabtree, 2004 ▸, 2018 ▸; Piccirilli *et al.*, 2020 ▸; Albrecht & van Koten, 2001 ▸). There are several pincer-ligand varieties in the literature ranging from those featuring both symmetric and non-symmetric flanking arms, P-, N-, O-, S- and C- donor sites, as well as neutral, mono, di- and trianionic systems. Monoanionic, carbon-centered (*e.g*., from phen­yl) ligands with P- or N-donors at the flanking arms are more common among pincers (Peris & Crabtree, 2018 ▸). These tridentate ligands are particularly inter­esting for their ability to preferentially occupy the *meridional* coordination sites on a metal ion.

**Figure 1:**
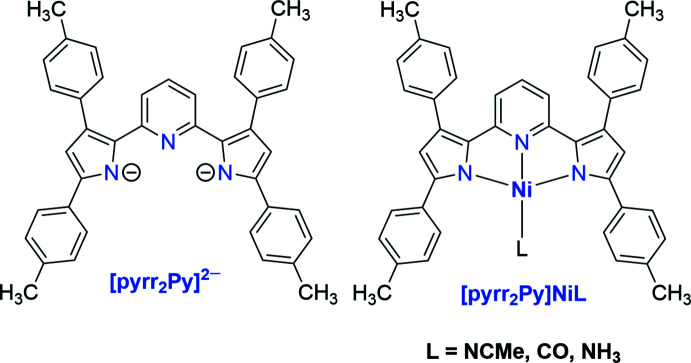
The dianionic, pyridine dipyrrolide pincer ligand [pyrr_2_Py]^2−^ and the nickel(II) complexes.

We have been working on tridentate, nitro­gen-based ligands such as tris­(pyrazol­yl)borates with a preference for *facial* coordination for several years (Dias & Lovely, 2008 ▸; Dias *et al.*, 1995 ▸, 1996 ▸; Dias & Lu, 1995 ▸). This paper describes results from our efforts to expand the ligand repertoire to include tridentate ligands with a preference for *meridional* geometry (Adiraju *et al.*, 2020 ▸) at transition-metal ions in our laboratory. In particular, we describe the synthesis and use of a pyridine dipyrrolide pincer ligand bearing tolyl substituents, (Pramanik *et al.*, 2014 ▸; Pramanik, 2015 ▸) and its chemistry with nickel(II) featuring CO, NH_3_ and NCMe mol­ecules (Fig. 1 ▸). The pyridine dipyrrolide is a particularly attractive ligand framework, as several examples of pyridine dipyrrolide pincers with different substituents on the ligand backbone (*e.g*., Me, *t*-Bu, Ph, Mes) are known and have already been successfully used with both early and late transition-metal ions such as Ti (Zhang *et al.*, 2016 ▸), Zr (Zhang *et al.*, 2016 ▸, 2020 ▸), Cr (Gowda *et al.*, 2018 ▸), Mo (Gowda *et al.*, 2018 ▸), Fe (Sorsche *et al.*, 2020 ▸; Hakey *et al.*, 2019 ▸), Co (Grant *et al.*, 2016 ▸), Pt (Komine *et al.*, 2014 ▸), Pd (Yadav *et al.*, 2018 ▸) and Zn (Komine *et al.*, 2014 ▸) to form well-defined metal complexes.
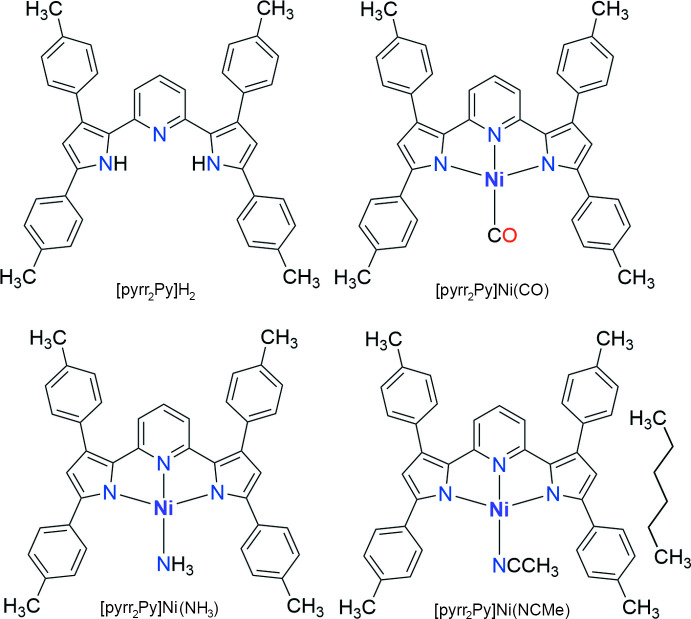


## Structural commentary   

**Figure 2:**
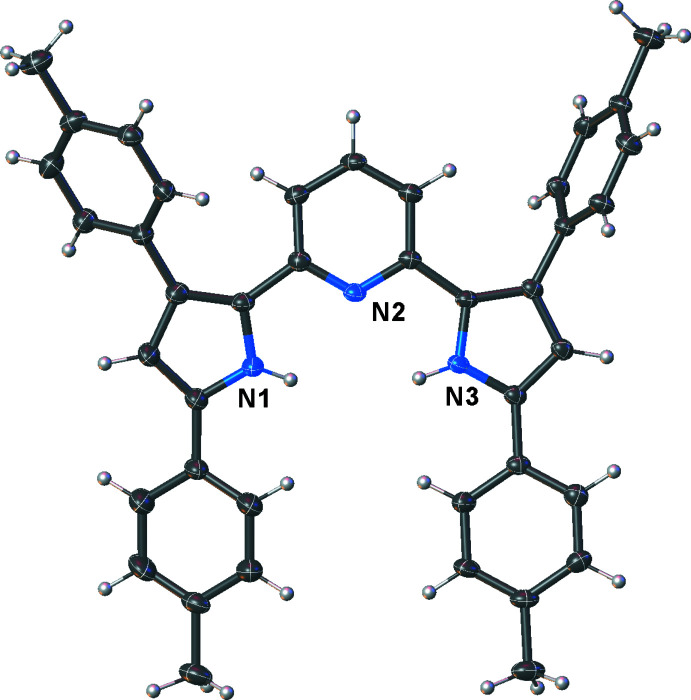
Mol­ecular structure of [pyrr_2_Py]H_2_ with displacement ellipsoids drawn at the 50% probability level.

**Figure 3:**
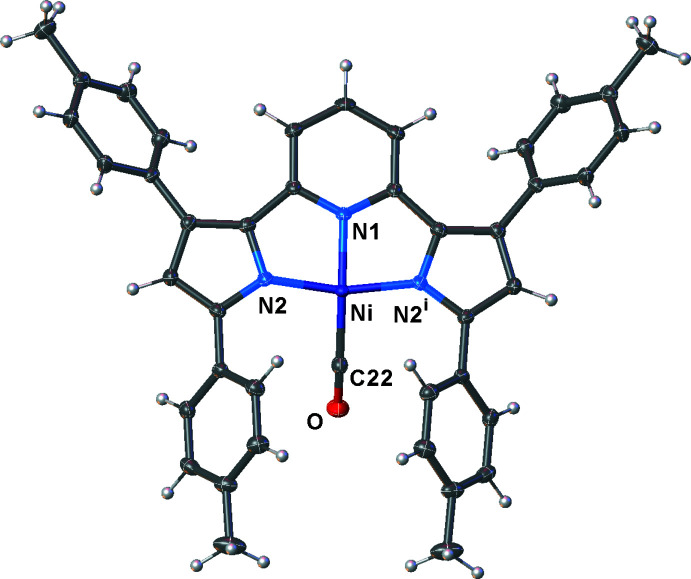
Mol­ecular structure of [pyrr_2_Py]Ni(CO) with displacement ellipsoids drawn at the 50% probability level. Symmetry code: (i) 1 + *x*, 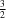 − *y*, z.

The free ligand [pyrr_2_Py]H_2_ is monomeric and crystallizes with both pyrrole nitro­gen atoms facing the center of the coord­ination pit, well situated for metal-ion coordination (Fig. 2 ▸). This is different from the structure observed with the *t*-butyl substituted pincer analog (VIWSOL; Komine *et al.*, 2014 ▸) in which one pyrrole N-H bond is directed outward to form a hydrogen bond with a lattice aceto­nitrile mol­ecule. The pyrrole and pyridine moieties are essentially coplanar. The nickel(II) carbonyl complex [pyrr_2_Py]Ni(CO) was synthesized from the *in situ-*generated potassium salt K_2_[pyrr_2_Py] and Ni(OTf)_2_ in the presence of carbon monoxide. The important CO stretch of this mol­ecule is observed at 2101 cm^−1^, which is only slightly lower than that of free CO (2143 cm^−1^), indicating relatively weak Ni→CO π-backbonding. The nickel(I) tris­(pyrazol­yl)borate complex [HB(3-Ph,5-MePz)_3_]Ni(CO) for comparison displays its CO stretch at 2003 cm^−1^ (Abubekerov *et al.*, 2016 ▸). The X-ray crystal structure shows that the pincer complex [pyrr_2_Py]Ni(CO) is a monomeric, square-planar complex (Fig. 3 ▸). The carbonyl moiety sits above the ligand plane, as is evident from the N1—Ni—C22 angle of 160.41 (13)°. The Ni—C22 distance of 1.809 (3) Å is significantly longer than the corresponding distance of 1.766 (4) Å in [HB(3-Ph,5-MePz)_3_]Ni(CO), which is a tetra­hedral nickel complex (ENUROW; Abubekerov *et al.*, 2016 ▸). The Ni—N(pyrr) (pyrr = pyrrolide) distances of 1.8667 (18) and 1.8666 (18) Å are not significantly different from the Ni—N(pyridine) separation of 1.853 (3) Å.

**Figure 4:**
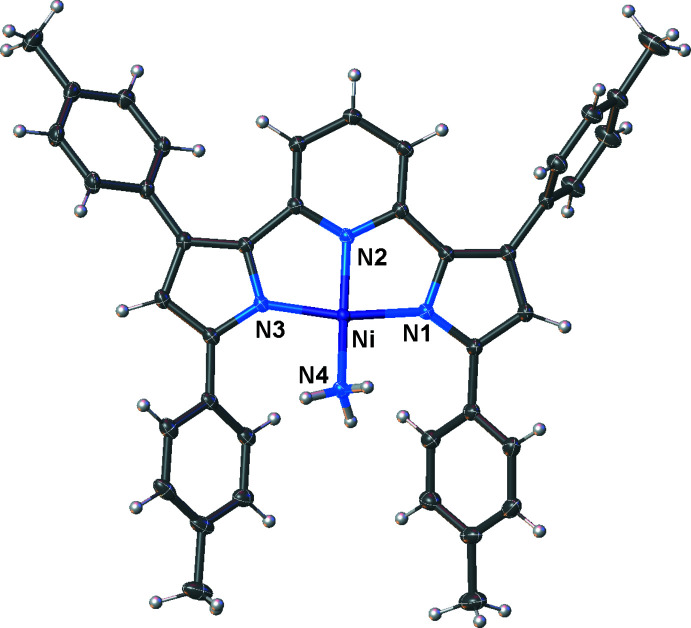
Mol­ecular structure of [pyrr_2_Py]Ni(NH_3_) with displacement ellipsoids drawn at the 50% probability level.

Compounds [pyrr_2_Py]Ni(NH_3_) and [pyrr_2_Py]Ni(NCMe) are also four-coordinate nickel(II) complexes with square-planar metal sites (Figs. 4 ▸ and 5 ▸, respectively). They have all nitro­gen coordination spheres at nickel, but with an inter­esting variety of donor sites ranging from *sp* to *sp* ^3^-hybridized nitro­gen atoms, as well as neutral and formally anionic N-centers. Both the NH_3_ and NCMe ligands bend out of the [pyrr_2_Py] ligand plane as evident from the N2—Ni—N4 angles of 162.16 (5) and 168.09 (10)°, respectively, for the two complexes. The Ni—N1 and Ni—N3 bond distances of [pyrr_2_Py]Ni(NH_3_) are 1.8858 (10) and 1.8876 (10) Å, respectively. These values are marginally smaller than the corresponding distances of [pyrr_2_Py]Ni(NCMe) [1.896 (2) and 1.906 (2) Å]. The Ni—N2 distances (to the pyridine moieties) at 1.8490 (10) and 1.846 (2) Å are similar in the two adducts, but they are both much shorter than the Ni—N(pyrr) distances noted above. The Ni—N bond distance to the NH_3_ and NCMe ligands in [pyrr_2_Py]Ni(NH_3_) and [pyrr_2_Py]Ni(NCMe) are 1.9291 (11) and 1.861 (2) Å, respectively. These are bonds to *sp* ^3^ and *sp-*hybridized nitro­gen sites, respectively, and therefore the longer distance for the former is not unusual. Four-coordinate nickel–ammonia complexes are rare and there is one example in the CSD (PEWROZ; Tapper *et al.*, 1993 ▸), and that has an Ni—N(H_3_) distance of 1.912 Å.

## Supra­molecular features   

**Figure 6:**
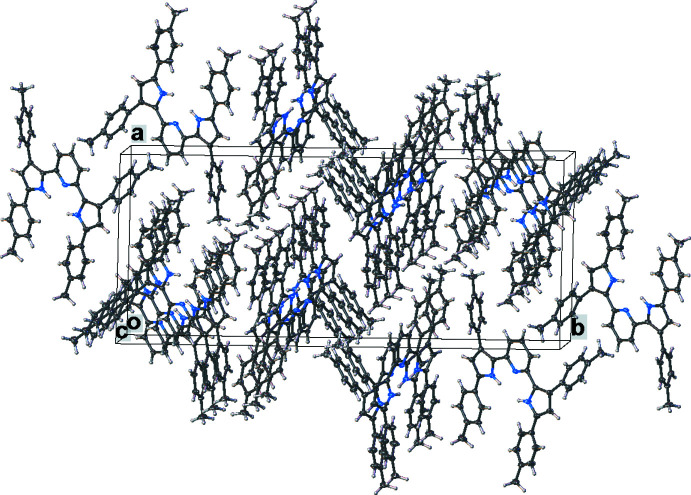
The crystal packing of [pyrr_2_Py]H_2_.

**Figure 7:**
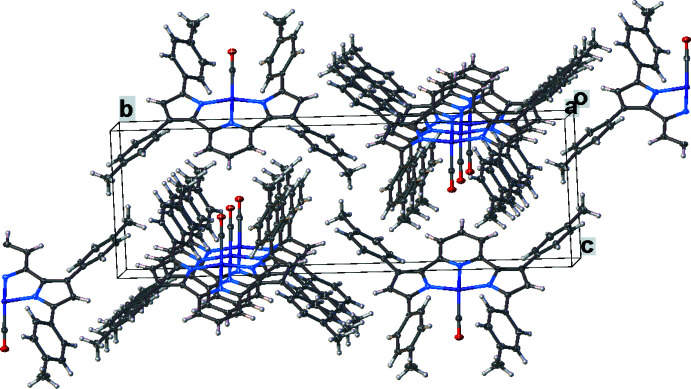
The crystal packing of [pyrr_2_Py]Ni(CO).

**Figure 8:**
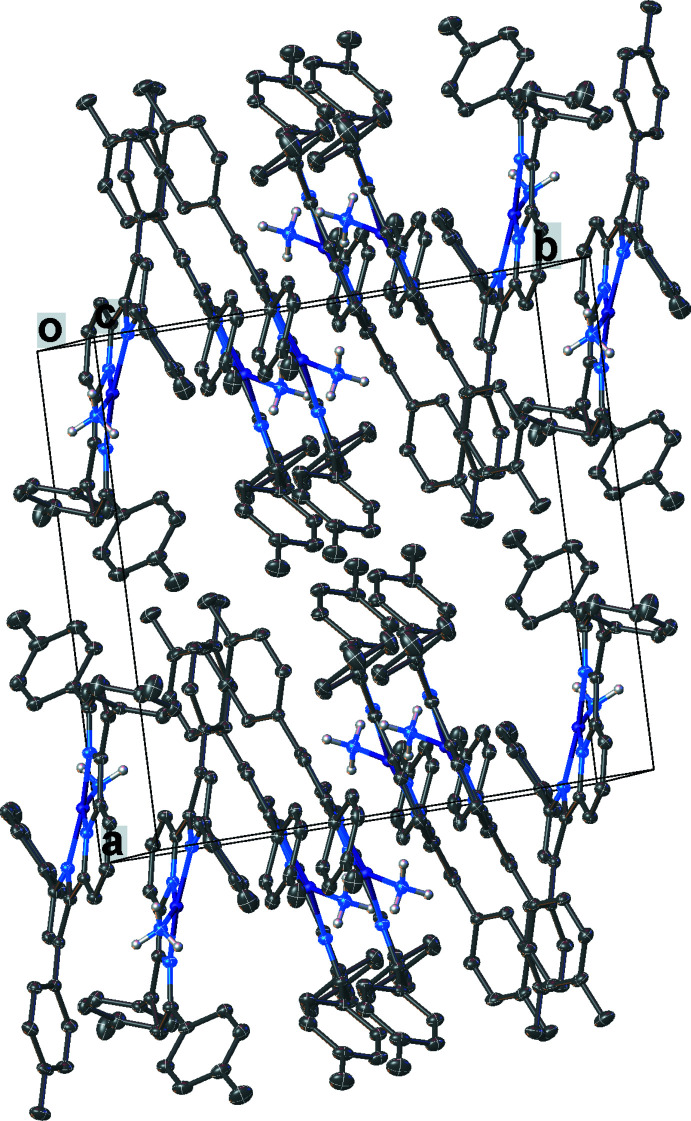
The crystal packing of [pyrr_2_Py]Ni(NH_3_). Hydrogen atoms except those on ammonia have been omitted for clarity.

**Figure 9:**
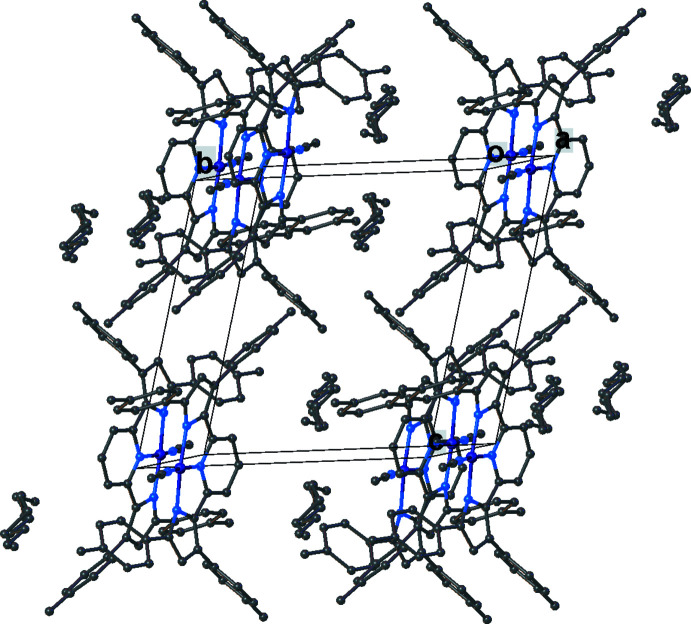
The crystal packing of [pyrr_2_Py]Ni(NCMe). Hydrogen atoms have been omitted for clarity.

Important inter­mol­ecular contacts and a packing diagram of [pyrr_2_Py]H_2_ are shown in Fig. 6 ▸ and Fig. S1 in the supporting information. Neighboring mol­ecules of [pyrr_2_Py]H_2_ show π–π contacts between pyrrole and pyridine groups (the closest separation is 3.21 Å) as well as C(arene)—H⋯arene contacts. The complex [pyrr_2_Py]Ni(CO) does not show extensive inter­molecular inter­actions apart from NiCO⋯H—C(arene) contacts between the carbonyl moieties and hydrogen atoms of neighboring arene as illustrated in Fig. 7 ▸ and Fig. S2. In the structure of [pyrr_2_Py]Ni(NH_3_), the arene groups inter­act with neighboring mol­ecules *via* the ammonia hydrogen atoms (see Fig. 8 ▸ and Fig. S3). In [pyrr_2_Py]Ni(NCMe), the hexane mol­ecules in the lattice occupy regions surrounded by tolyl substituents. The major inter­molecular inter­actions are between arenes and the hydrogen atoms of the aceto­nitrile moieties. The resulting packing diagram is shown in Fig. 9 ▸ and Fig. S4. 

## Database survey   

A search of the Cambridge Structural Database for related pyridine dipyrrolide complexes involving transition-metal ions revealed 38 hits involving ligands with different alkyl or aryl substituents (CSD Version 5.41, Update 2, May 2020; Groom *et al.*, 2016 ▸). No nickel pyridine dipyrrolide complexes have been reported thus far. Perhaps the most closely related compounds are the four-coordinate platinum (VIWSIF; Komine *et al.*, 2014 ▸), palladium (XIKKIO, XIKKOU; Yadav *et al.*, 2018 ▸) and zinc (VIWSIF; Komine *et al.*, 2014 ▸) complexes featuring all-nitro­gen coordination spheres at the metal. In addition, there are ten hits for related free ligands. Most of them, however, are different solvates of the same ligand system.

## Synthesis and crystallization   

All experiments were done under a purified nitro­gen atmosphere with standard Schlenk techniques. Solvents were purchased from commercial sources and purified using an Innovative Technology SPS-400 PureSolv solvent-drying system or distilled over conventional drying agents and degassed by the freeze–pump–thaw method three times prior to use. All other chemicals needed were obtained from commercial vendors. Glassware was oven dried at 150°C overnight. The NMR spectra were recorded at 25°C on JEOL Eclipse 500 and 300 spectrometers (^1^H: 500.16 MHz or 300.53 MHz). Proton chemical shifts are reported in ppm *versus* Me_4_Si. Infrared spectra were taken on a JASCO FT–IR 410 spectrometer.

**Synthesis of 2,6-bis­(3,5-ditolyl-2-pyrrol­yl)pyridine, [pyrr_2_Py]H_2_:**

1,3-Bis(4-tol­yl)-2-propen-1-one (chalcone) was prepared following a literature procedure (Yang *et al.*, 2005 ▸) from tolu­aldehyde and 4-methyl­aceto­phenone. Then the chalcone (1.75 g, 7.4 mmol) was reacted with 2,6-pyridine­dicarbaldehyde (0.50 g, 3.7 mmol), 3-benzyl-5-(-hy­droxy­eth­yl)-4-methyl­thia­zolium chloride (0.20 g, 0.74 mmol) and sodium *t*-butoxide (0.57 g, 0.74 mmol) in ethanol at reflux for 24 h to form a brown suspension. Water was added and the mixture was extracted with chloro­form. The chloro­form was removed to obtain 2,6-bis­(2,4-ditolyl-1,4-dioxobut­yl)pyridine. This was purified further by rinsing with hexane to get an orange solid. The inter­mediate ketone was reacted with NH_4_OAc (2.8 g, 37 mmol) in ethanol at reflux for 24 h. Water was added and the yellow solid was filtered and washed with water. Then the crude product was suspended in 10 mL of ethanol and refluxed at 373 K for 7 h to obtain 2,6-bis­(3,5-ditolyl-2-pyrrol­yl)pyridine, [pyrr_2_Py]H_2_ as a yellow solid (yield 64%). ^1^H NMR (CDCl_3_, 500.16 MHz, 298 K): δ 2.38 (*s*, 12H, CH_3_) 6.57 (*m*, 2H), 7.02 (*d*, *J* = 8.05, 2H), 7.17–7.22 (*m*, 9H), 7.38 (*d*, 4H), 7.47 (*d*, *J* = 8 Hz, 4H), 9.56 (2H, NH).

**Synthesis of [pyrr_2_Py]Ni(NCCH_3_):**

A solid sample of the ligand 2,6-bis­(3,5-ditolyl-2-pyrrol­yl)pyridine ([pyrr_2_Py]H_2_; 0.10 g, 0.175 mmol) and KH (0.021 g, 0.525 mmol) were placed in a 50 mL Schlenk flask. THF (*ca* 10 mL) was added to the mixture at room temperature and then refluxed for 1.5 h. It was allowed to cool down to room temperature and filtered through a Celite pad, which was then washed with 5 mL of THF. The filtrate was collected and added to Ni(OTf)_2_ (0.062 g, 0.175 mmol) in 10 mL of THF and stirred overnight at room temperature. The volatile materials were removed under reduced pressure and the residue was extracted into ether and filtered. Ether was removed under vacuum and 10 mL of aceto­nitrile were added. After 1 h, it was filtered, and the filtrate was concentrated to 4 mL. Finally, hexane was layered above the aceto­nitrile and allowed to diffuse slowly into aceto­nitrile solution at room temperature, producing brown crystals of [pyrr_2_Py]Ni(CH_3_CN) (yield 34%). ^1^H NMR (CDCl_3_, 500.16 MHz, 298 K): δ 0.738 (*s*, 3H, CH_3_) 2.32 (*s*, 6H, C*H*_3_), 2.37 (*s*, 6H, CH_3_) 6.06 (*s*, 2H), 6.60 (*d*, *J* = 8 Hz, 2H), 7.04 (*t*, *J* = 8 Hz, 1H), 7.15 (*m*, 8H), 7.36 (*d*, *J* = 8 Hz, 4H), 7.62 (*d*, *J* = 8.05 Hz, 4H).

**Synthesis of [pyrr_2_Py]Ni(CO):**

A solid sample of the ligand 2,6-bis­(3,5-ditolyl-2-pyrrol­yl)pyridine ([pyrr_2_Py]H_2_) (0.10 g, 0.175 mmol) and KH (0.021 g, 0.525 mmol) were placed in a 50 mL Schlenk flask. THF (*ca* 10 mL) was added to the mixture at room temperature and then refluxed for 1.5 h. It was allowed to cool down to room temperature and filtered through a Celite pad, which was then washed with 5 mL of THF. The filtrate was added to Ni(OTf)_2_ (0.062 g, 0.175 mmol) in 10 mL of THF and stirred overnight at room temperature. Then THF was removed and the residue was extracted into ether. Then anhydrous carbon monoxide gas was passed through the ether solution for 20 minutes at 273 K. After stirring for 1 h, the solution was filtered, and the volume of the solution was decreased to 4 mL. Red crystals of [pyrr_2_Py]Ni(CO) were observed after keeping the solution in a 253 K freezer for 3 d (yield 24%). ^1^H NMR (CDCl_3_, 500.16 MHz, 298 K): δ 2.37 (*s*, 6H, C*H*_3_), 2.38 (*s*, 6H, CH_3_) 6.21 (*s*, 2H), 6.77 (*d*, *J* = 7.45 Hz, 2H), 7.02 (*t*, *J* = 8 Hz, 1H), 7.21 (*m*, 8H), 7.38 (*d*, *J* = 7.5 Hz, 4H), 7.47 (*d*, *J* = 8.05 Hz, 4H). ^13^C{^1^H} NMR (CDCl_3_, 125.77 MHz, 298 K, selected): δ 174.4 (CO). IR (crystals, ATR, selected band) cm^−1^: 2101 (CO).

**Synthesis of [pyrr_2_Py]Ni(NH_3_):**

A solid sample of the ligand 2,6-bis­(3,5-ditolyl-2-pyrrol­yl)pyridine ([pyrr_2_Py]H_2_) (0.10 g, 0.175 mmol) and KH (0.021 g, 0.525 mmol) were placed in a 50 mL Schlenk flask. THF (*ca* 10 mL) was added to the mixture at room temperature and then refluxed for 1.5 h. It was allowed to cool down to room temperature and filtered through a Celite pad, which was then washed with 5 mL of THF. The filtrate was added to Ni(OTf)_2_ (0.062 g, 0.175 mmol) in 10 mL of THF and stirred overnight at room temperature. Then THF was removed and the residue was extracted into ether. Then anhydrous ammonia gas was passed through the ether solution for 20 minutes at 273 K. After stirring for 1 h, the solution was filtered, and the volume of the solution was decreased to 4 mL. Red crystals of [pyrr_2_Py]Ni(NH_3_) were formed after keeping the solution in a 253 K freezer for 3 d (yield 54%). ^1^H NMR (CDCl_3_, 500.16 MHz, 298 K): δ 0.49 (*s*, 3H, NH_3_) 2.35 (*s*, 6H, C*H*_3_), 2.38 (*s*, 6H, CH_3_) 6.08 (*s*, 2H), 6.63 (*d*, *J* = 8 Hz, 2H), 7.05 (*t*, *J* = 8.05 Hz, 1H), 7.19 (*m*, 8H), 7.36 (*d*, *J* = 8.05, 4H), 7.62 (*d*, *J* = 7.45 Hz, 4H). IR (crystals, ATR, selected bands) cm^−1^: 3310, 3360 (NH).

## Refinement   

Crystal data, data collection and structure refinement details for [pyrr_2_Py]H_2_, [pyrr_2_Py]Ni(CO), [pyrr_2_Py]Ni(NH_3_) and [pyrr_2_Py]Ni(NCMe)·hexane are summarized in Table 1 ▸. Non-H atoms were refined with anisotropic displacement parameters. Hydrogen atoms, except for the N—H hydrogen atoms, were placed in calculated positions using riding models, and refined riding on their parent atoms with C—H = 0.95 Å and *U*_iso_(H) = 1.2*U*_eq_(C) for aromatic hydrogen atoms, C—H = 0.99 Å and *U*_iso_(H) = 1.2*U*_eq_(C) for methyl­ene hydrogen atoms (of hexa­ne), and C—H = 0.98 Å with *U*_iso_(H) = 1.5*U*_eq_(C) for methyl hydrogen atoms. The N—H hydrogen atoms of [pyrr_2_Py]H_2_ and [pyrr_2_Py]Ni(NH_3_) were obtained from a difference-Fourier map and refined freely. The nickel carbonyl complex [pyrr_2_Py]Ni(CO) is located on a plane of symmetry containing the Ni–CO moiety but perpendicular to the [pyrr_2_Py] ligand plane, and consequently only a half is contained in the asymmetric unit. The complex [pyrr_2_Py]Ni(NCCH_3_) crystallizes with a mol­ecule of hexane, which was disordered over two sites [with refined occupancy rates of 77.9 (5)% and 22.1 (5)%]. C—C bond distances were restrained to a target value of 1.53 (2) Å (DFIX restraint of *SHELXL*), 1,3 C⋯C distances were restrained to be similar to each other (SADI restraint of *SHELXL*, esd = 0.04 Å), and *U* ^ij^ components of ADPs were restrained to be similar for atoms closer to each other than two Å (SIMU restraint of *SHELXL*, esd = 0.02 Å^2^ for terminal atoms and 0.01 Å^2^ for all others).

## Supplementary Material

Crystal structure: contains datablock(s) pyrr2PyH2, pyrr2PyNiCO, pyrr2PyNiNH3, pyrr2PyNiCNMe. DOI: 10.1107/S2056989020013341/zl2799sup1.cif

Structure factors: contains datablock(s) pyrr2PyH2. DOI: 10.1107/S2056989020013341/zl2799pyrr2PyH2sup2.hkl

Structure factors: contains datablock(s) pyrr2PyNiCO. DOI: 10.1107/S2056989020013341/zl2799pyrr2PyNiCOsup3.hkl

Structure factors: contains datablock(s) pyrr2PyNiNH3. DOI: 10.1107/S2056989020013341/zl2799pyrr2PyNiNH3sup4.hkl

Structure factors: contains datablock(s) pyrr2PyNiCNMe. DOI: 10.1107/S2056989020013341/zl2799pyrr2PyNiCNMesup5.hkl

CCDC references: 2035500, 2035499, 2035498, 2035497

Additional supporting information:  crystallographic information; 3D view; checkCIF report

## Figures and Tables

**Figure 5:**
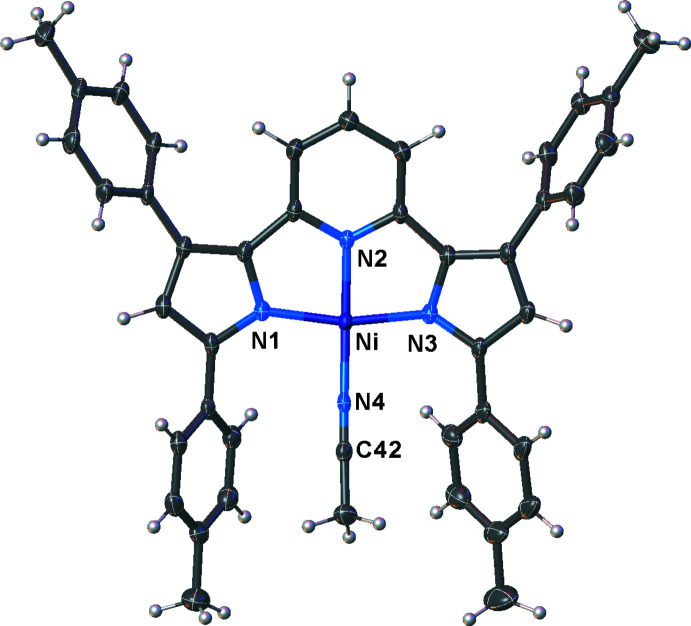
Mol­ecular structure of [pyrr_2_Py]Ni(NCMe) with displacement ellipsoids drawn at the 50% probability level. A disordered hexane mol­ecule has been omitted for clarity.

**Table 1:** Experimental details

	[pyrr_2_PyH_2_]	[pyrr_2_Py]Ni(CO)	[pyrr_2_Py]Ni(NH_3_)	[pyrr_2_Py]Ni(NCMe)
Crystal data				
Chemical formula	C_41_H_35_N_3_	[Ni(C_41_H_33_N_3_)(CO)]	[Ni(C_41_H_33_N_3_)(NH_3_)]	[Ni(C_41_H_33_N_3_)(C_2_H_3_N)]
*M*_r_	569.72	654.42	643.45	753.64
Crystal system, space group	Monoclinic, *P*2_1_/*c*	Monoclinic, *P*12_1_/*m*1	Monoclinic, *P*2_1_/*c*	Triclinic, *P* 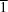
Temperature (K)	100	100	100	100
*a*, *b*, *c* (Å)	14.8940 (15), 35.155 (4), 5.9513 (6)	6.6482 (4), 27.1709 (18), 9.1322 (6)	15.9773 (6), 14.9441 (5), 14.3238 (5)	11.2735 (16), 14.1802 (19), 14.688 (2)
α, β, γ (°)	90, 100.987 (2), 90	90, 101.0700 (12), 90	90, 107.8140 (8), 90	67.162 (2), 68.881 (2), 80.665 (2)
*V* (Å^3^)	3059.0 (5)	1618.92 (18)	3256.1 (2)	2018.0 (5)
*Z*	4	2	4	2
Radiation type	Mo *K*α	Mo *K*α	Mo *K*α	Mo *K*α
μ (mm^−1^)	0.07	0.64	0.63	0.52
Crystal size (mm)	0.28 × 0.18 × 0.12	0.36 × 0.27 × 0.05	0.46 × 0.41 × 0.14	0.20 × 0.12 × 0.09

Data collection				
Diffractometer	Bruker D8 Quest with a Photon 100 CMOS detector	Bruker D8 Quest with a Photon 100 CMOS detector	Bruker D8 Quest with a Photon 100 CMOS detector	Bruker APEXII CCD
Absorption correction	Multi-scan (*SADABS*; Bruker, 2016 ▸)	Multi-scan (*SADABS*; Bruker, 2016 ▸)	Multi-scan (*SADABS*; Bruker, 2016 ▸)	Multi-scan (*SADABS*; Bruker, 2016 ▸)
*T*_min_, *T*_max_	0.341, 0.431	0.859, 1.000	0.858, 0.967	0.686, 0.899
No. of measured, independent and observed [*I* > 2σ(*I*)] reflections	31843, 7596, 5005	18888, 4080, 3280	48278, 9931, 8502	21869, 9994, 7327
*R*_int_	0.076	0.057	0.026	0.058
(sin θ/λ)_max_ (Å^−1^)	0.669	0.667	0.714	0.667

Refinement				
*R*[*F* ^2^ > 2σ(*F* ^2^)], *wR*(*F* ^2^), *S*	0.076, 0.182, 1.06	0.050, 0.119, 1.07	0.036, 0.098, 1.05	0.068, 0.193, 1.00
No. of reflections	7596	4080	9931	9994
No. of parameters	409	222	431	551
No. of restraints	0	0	0	178
H-atom treatment	H atoms treated by a mixture of independent and constrained refinement	H-atom parameters constrained	H atoms treated by a mixture of independent and constrained refinement	H-atom parameters constrained
Δρ_max_, Δρ_min_ (e Å^−3^)	0.40, −0.25	0.67, −0.33	0.51, −0.26	1.75, −0.93

Computer programs: *APEX3* and *SAINT* (Bruker, 2016 ▸), *SHELXT* (Sheldrick, 2015*b* ▸), *SHELXL* (Sheldrick, 2015*a* ▸) and *OLEX2* (Dolomanov *et al.*, 2009 ▸).

## supplementary crystallographic information

### 2,6-Bis[3,5-bis(4-methylphenyl)pyrrol-2-yl]pyridine (pyrr2PyH2) Crystal data

**Table:**

C_41_H_35_N_3_	*F*(000) = 1208
*M**_r_* = 569.72	*D*_x_ = 1.237 Mg m^−^^3^
Monoclinic, *P*2_1_/*c*	Mo *K*α radiation, λ = 0.71073 Å
*a* = 14.8940 (15) Å	Cell parameters from 6014 reflections
*b* = 35.155 (4) Å	θ = 3.0–28.0°
*c* = 5.9513 (6) Å	µ = 0.07 mm^−^^1^
β = 100.987 (2)°	*T* = 100 K
*V* = 3059.0 (5) Å^3^	Plates, yellow
*Z* = 4	0.28 × 0.18 × 0.12 mm

### 2,6-Bis[3,5-bis(4-methylphenyl)pyrrol-2-yl]pyridine (pyrr2PyH2) Data collection

**Table:**

Bruker D8 Quest with a Photon 100 CMOS detector diffractometer	7596 independent reflections
Radiation source: sealed tube	5005 reflections with *I* > 2σ(*I*)
Curved-graphite monochromator	*R*_int_ = 0.076
Detector resolution: 8 pixels mm^-1^	θ_max_ = 28.4°, θ_min_ = 2.9°
φ and ω scans	*h* = −19→19
Absorption correction: multi-scan (SADABS; Bruker, 2016)	*k* = −46→46
*T*_min_ = 0.341, *T*_max_ = 0.431	*l* = −7→7
31843 measured reflections	

### 2,6-Bis[3,5-bis(4-methylphenyl)pyrrol-2-yl]pyridine (pyrr2PyH2) Refinement

**Table:**

Refinement on *F*^2^	Primary atom site location: dual
Least-squares matrix: full	Secondary atom site location: difference Fourier map
*R*[*F*^2^ > 2σ(*F*^2^)] = 0.076	Hydrogen site location: mixed
*wR*(*F*^2^) = 0.182	H atoms treated by a mixture of independent and constrained refinement
*S* = 1.06	*w* = 1/[σ^2^(*F*_o_^2^) + (0.0644*P*)^2^ + 2.7619*P*] where *P* = (*F*_o_^2^ + 2*F*_c_^2^)/3
7596 reflections	(Δ/σ)_max_ < 0.001
409 parameters	Δρ_max_ = 0.40 e Å^−^^3^
0 restraints	Δρ_min_ = −0.25 e Å^−^^3^

### 2,6-Bis[3,5-bis(4-methylphenyl)pyrrol-2-yl]pyridine (pyrr2PyH2) Special details

**Table:**

Geometry. All esds (except the esd in the dihedral angle between two l.s. planes) are estimated using the full covariance matrix. The cell esds are taken into account individually in the estimation of esds in distances, angles and torsion angles; correlations between esds in cell parameters are only used when they are defined by crystal symmetry. An approximate (isotropic) treatment of cell esds is used for estimating esds involving l.s. planes.

### 2,6-Bis[3,5-bis(4-methylphenyl)pyrrol-2-yl]pyridine (pyrr2PyH2) Fractional atomic coordinates and isotropic or equivalent isotropic displacement parameters (Å^2^)

**Table:**

	*x*	*y*	*z*	*U*_iso_*/*U*_eq_	
N1	0.31778 (13)	0.41078 (6)	0.3752 (4)	0.0269 (5)	
H1	0.3085 (17)	0.3918 (8)	0.284 (5)	0.023 (7)*	
N2	0.15143 (12)	0.38454 (5)	0.2069 (3)	0.0220 (4)	
N3	0.15725 (13)	0.34057 (6)	−0.1524 (3)	0.0218 (4)	
H3	0.201 (2)	0.3567 (8)	−0.090 (5)	0.035 (8)*	
C1	0.39793 (16)	0.42861 (7)	0.4672 (4)	0.0284 (6)	
C2	0.37801 (16)	0.45430 (8)	0.6255 (5)	0.0309 (6)	
H2	0.420528	0.470845	0.716611	0.037*	
C3	0.28267 (16)	0.45185 (7)	0.6295 (4)	0.0268 (5)	
C4	0.24695 (15)	0.42390 (7)	0.4728 (4)	0.0239 (5)	
C5	0.48235 (16)	0.41980 (7)	0.3826 (5)	0.0298 (6)	
C6	0.47946 (18)	0.40299 (9)	0.1714 (5)	0.0411 (7)	
H6	0.421944	0.397431	0.077399	0.049*	
C7	0.55943 (19)	0.39409 (9)	0.0944 (6)	0.0422 (7)	
H7	0.555510	0.382415	−0.051101	0.051*	
C8	0.64462 (17)	0.40191 (8)	0.2254 (5)	0.0339 (6)	
C9	0.64744 (19)	0.41900 (10)	0.4340 (6)	0.0486 (8)	
H9	0.705182	0.424893	0.525887	0.058*	
C10	0.56826 (18)	0.42799 (9)	0.5151 (6)	0.0456 (8)	
H10	0.572601	0.439699	0.660598	0.055*	
C11	0.73140 (18)	0.39198 (9)	0.1421 (6)	0.0419 (7)	
H11A	0.778705	0.410843	0.199197	0.063*	
H11B	0.719407	0.391943	−0.025612	0.063*	
H11C	0.752380	0.366705	0.198732	0.063*	
C12	0.23419 (16)	0.47692 (7)	0.7652 (4)	0.0257 (5)	
C13	0.27181 (17)	0.48578 (7)	0.9915 (4)	0.0304 (6)	
H13	0.329417	0.475331	1.059455	0.036*	
C14	0.22680 (19)	0.50962 (8)	1.1203 (5)	0.0327 (6)	
H14	0.253710	0.514926	1.274969	0.039*	
C15	0.14287 (18)	0.52581 (7)	1.0254 (5)	0.0299 (6)	
C16	0.10546 (17)	0.51734 (7)	0.7991 (5)	0.0286 (6)	
H16	0.048060	0.528006	0.731306	0.034*	
C17	0.15026 (16)	0.49357 (7)	0.6694 (5)	0.0265 (5)	
H17	0.123589	0.488589	0.514138	0.032*	
C18	0.0944 (2)	0.55256 (8)	1.1610 (5)	0.0396 (7)	
H18A	0.106102	0.578898	1.120927	0.059*	
H18B	0.117200	0.548580	1.324950	0.059*	
H18C	0.028499	0.547566	1.125210	0.059*	
C19	0.15686 (15)	0.40612 (7)	0.3944 (4)	0.0226 (5)	
C20	0.08401 (16)	0.40989 (7)	0.5100 (4)	0.0247 (5)	
H20	0.090314	0.424150	0.647765	0.030*	
C21	0.00241 (16)	0.39217 (7)	0.4173 (4)	0.0250 (5)	
H21	−0.048992	0.394936	0.489085	0.030*	
C22	−0.00481 (16)	0.37054 (7)	0.2219 (4)	0.0245 (5)	
H22	−0.061207	0.358902	0.155506	0.029*	
C23	0.07227 (15)	0.36606 (6)	0.1232 (4)	0.0205 (5)	
C24	0.07687 (15)	0.34119 (7)	−0.0706 (4)	0.0215 (5)	
C25	0.01808 (15)	0.31582 (7)	−0.2046 (4)	0.0217 (5)	
C26	0.06570 (15)	0.30020 (7)	−0.3677 (4)	0.0235 (5)	
H26	0.042322	0.281964	−0.481713	0.028*	
C27	0.15233 (15)	0.31619 (7)	−0.3319 (4)	0.0219 (5)	
C28	−0.07829 (15)	0.30748 (7)	−0.1857 (4)	0.0220 (5)	
C29	−0.09980 (16)	0.28944 (7)	0.0044 (4)	0.0258 (5)	
H29	−0.051897	0.280648	0.121771	0.031*	
C30	−0.18999 (17)	0.28401 (7)	0.0264 (4)	0.0279 (5)	
H30	−0.202766	0.271797	0.159191	0.033*	
C31	−0.26155 (16)	0.29607 (7)	−0.1417 (5)	0.0279 (6)	
C32	−0.24067 (16)	0.31310 (7)	−0.3364 (5)	0.0300 (6)	
H32	−0.288788	0.321051	−0.455786	0.036*	
C33	−0.15012 (16)	0.31866 (7)	−0.3587 (4)	0.0264 (5)	
H33	−0.137268	0.330214	−0.493386	0.032*	
C34	−0.35951 (18)	0.29081 (9)	−0.1141 (6)	0.0425 (7)	
H34A	−0.376282	0.311221	−0.017785	0.064*	
H34B	−0.365963	0.266169	−0.041886	0.064*	
H34C	−0.399856	0.291641	−0.264779	0.064*	
C35	0.23013 (15)	0.31055 (7)	−0.4467 (4)	0.0224 (5)	
C36	0.21959 (17)	0.29117 (7)	−0.6546 (4)	0.0258 (5)	
H36	0.161229	0.281478	−0.722971	0.031*	
C37	0.29340 (17)	0.28587 (7)	−0.7625 (4)	0.0274 (5)	
H37	0.284801	0.272442	−0.903541	0.033*	
C38	0.38005 (16)	0.29991 (7)	−0.6680 (4)	0.0287 (6)	
C39	0.39012 (16)	0.31946 (8)	−0.4625 (5)	0.0321 (6)	
H39	0.448292	0.329511	−0.395562	0.039*	
C40	0.31675 (16)	0.32462 (8)	−0.3528 (5)	0.0295 (6)	
H40	0.325632	0.337963	−0.211463	0.035*	
C41	0.46052 (19)	0.29329 (9)	−0.7846 (5)	0.0396 (7)	
H41A	0.505142	0.313831	−0.744108	0.059*	
H41B	0.439234	0.292851	−0.950850	0.059*	
H41C	0.489307	0.268885	−0.734267	0.059*	

### 2,6-Bis[3,5-bis(4-methylphenyl)pyrrol-2-yl]pyridine (pyrr2PyH2) Atomic displacement parameters (Å^2^)

**Table:**

	*U*^11^	*U*^22^	*U*^33^	*U*^12^	*U*^13^	*U*^23^
N1	0.0196 (10)	0.0316 (12)	0.0297 (12)	0.0012 (8)	0.0049 (8)	−0.0087 (10)
N2	0.0167 (9)	0.0257 (10)	0.0240 (11)	0.0030 (8)	0.0051 (8)	0.0003 (8)
N3	0.0155 (9)	0.0277 (11)	0.0224 (10)	0.0006 (8)	0.0041 (8)	−0.0005 (8)
C1	0.0197 (12)	0.0332 (14)	0.0315 (14)	0.0017 (10)	0.0025 (10)	−0.0049 (11)
C2	0.0203 (12)	0.0380 (14)	0.0322 (14)	0.0016 (10)	−0.0007 (10)	−0.0084 (12)
C3	0.0214 (11)	0.0311 (13)	0.0270 (13)	0.0054 (10)	0.0023 (10)	−0.0012 (11)
C4	0.0199 (11)	0.0271 (12)	0.0254 (13)	0.0047 (9)	0.0061 (9)	0.0001 (10)
C5	0.0203 (12)	0.0307 (14)	0.0385 (15)	0.0021 (10)	0.0062 (11)	−0.0067 (12)
C6	0.0221 (13)	0.0505 (18)	0.0509 (19)	−0.0024 (12)	0.0074 (12)	−0.0149 (15)
C7	0.0290 (14)	0.0495 (18)	0.0504 (19)	−0.0028 (12)	0.0130 (13)	−0.0179 (15)
C8	0.0218 (12)	0.0321 (14)	0.0497 (18)	0.0006 (10)	0.0117 (12)	−0.0001 (13)
C9	0.0199 (13)	0.060 (2)	0.063 (2)	−0.0009 (13)	0.0015 (13)	−0.0137 (17)
C10	0.0257 (14)	0.060 (2)	0.0500 (19)	−0.0005 (13)	0.0037 (13)	−0.0205 (16)
C11	0.0251 (14)	0.0444 (17)	0.060 (2)	−0.0001 (12)	0.0179 (13)	0.0017 (15)
C12	0.0230 (12)	0.0261 (13)	0.0282 (13)	0.0019 (9)	0.0056 (10)	−0.0037 (10)
C13	0.0276 (13)	0.0338 (14)	0.0286 (14)	0.0050 (10)	0.0023 (11)	−0.0023 (11)
C14	0.0402 (15)	0.0322 (14)	0.0260 (14)	0.0027 (11)	0.0071 (11)	−0.0039 (11)
C15	0.0355 (14)	0.0259 (13)	0.0317 (14)	0.0020 (10)	0.0147 (11)	0.0011 (11)
C16	0.0247 (12)	0.0254 (13)	0.0367 (15)	0.0031 (10)	0.0087 (11)	0.0009 (11)
C17	0.0225 (12)	0.0258 (13)	0.0309 (14)	−0.0005 (9)	0.0042 (10)	−0.0027 (10)
C18	0.0478 (17)	0.0346 (15)	0.0415 (17)	0.0094 (13)	0.0212 (14)	−0.0009 (13)
C19	0.0213 (11)	0.0215 (12)	0.0253 (13)	0.0028 (9)	0.0052 (9)	0.0015 (10)
C20	0.0257 (12)	0.0241 (12)	0.0263 (13)	0.0033 (9)	0.0098 (10)	−0.0005 (10)
C21	0.0226 (12)	0.0227 (12)	0.0333 (14)	0.0036 (9)	0.0141 (10)	0.0010 (10)
C22	0.0201 (11)	0.0255 (12)	0.0294 (13)	0.0007 (9)	0.0084 (10)	0.0020 (10)
C23	0.0184 (11)	0.0225 (11)	0.0213 (12)	0.0020 (9)	0.0054 (9)	0.0039 (9)
C24	0.0171 (10)	0.0271 (12)	0.0212 (12)	0.0026 (9)	0.0061 (9)	0.0018 (10)
C25	0.0185 (11)	0.0248 (12)	0.0216 (12)	0.0003 (9)	0.0032 (9)	0.0028 (10)
C26	0.0203 (11)	0.0276 (13)	0.0222 (12)	0.0000 (9)	0.0027 (9)	0.0013 (10)
C27	0.0176 (11)	0.0266 (12)	0.0211 (12)	0.0023 (9)	0.0029 (9)	0.0024 (10)
C28	0.0191 (11)	0.0225 (12)	0.0248 (12)	−0.0005 (9)	0.0053 (9)	−0.0024 (9)
C29	0.0228 (12)	0.0309 (13)	0.0234 (13)	−0.0007 (10)	0.0037 (10)	0.0013 (10)
C30	0.0306 (13)	0.0297 (13)	0.0259 (13)	−0.0060 (10)	0.0120 (11)	−0.0011 (10)
C31	0.0202 (12)	0.0278 (13)	0.0381 (15)	−0.0033 (9)	0.0114 (10)	−0.0069 (11)
C32	0.0201 (12)	0.0331 (14)	0.0360 (15)	0.0014 (10)	0.0034 (10)	0.0022 (11)
C33	0.0202 (11)	0.0322 (13)	0.0276 (13)	−0.0003 (10)	0.0071 (10)	0.0039 (11)
C34	0.0243 (14)	0.0534 (18)	0.0538 (19)	−0.0074 (12)	0.0179 (13)	−0.0046 (15)
C35	0.0195 (11)	0.0249 (12)	0.0231 (12)	0.0058 (9)	0.0048 (9)	0.0038 (10)
C36	0.0242 (12)	0.0270 (13)	0.0258 (13)	0.0018 (9)	0.0035 (10)	0.0027 (10)
C37	0.0302 (13)	0.0299 (13)	0.0234 (13)	0.0052 (10)	0.0082 (10)	−0.0003 (10)
C38	0.0221 (12)	0.0351 (14)	0.0311 (14)	0.0080 (10)	0.0110 (10)	0.0046 (11)
C39	0.0160 (11)	0.0453 (16)	0.0359 (15)	0.0023 (10)	0.0071 (10)	−0.0037 (12)
C40	0.0222 (12)	0.0386 (15)	0.0282 (14)	0.0013 (10)	0.0058 (10)	−0.0058 (11)
C41	0.0289 (14)	0.0569 (19)	0.0368 (16)	0.0090 (13)	0.0158 (12)	0.0023 (14)

### 2,6-Bis[3,5-bis(4-methylphenyl)pyrrol-2-yl]pyridine (pyrr2PyH2) Geometric parameters (Å, º)

**Table:**

N1—H1	0.86 (3)	C19—C20	1.398 (3)
N1—C1	1.367 (3)	C20—H20	0.9500
N1—C4	1.377 (3)	C20—C21	1.384 (3)
N2—C19	1.339 (3)	C21—H21	0.9500
N2—C23	1.355 (3)	C21—C22	1.377 (3)
N3—H3	0.89 (3)	C22—H22	0.9500
N3—C24	1.376 (3)	C22—C23	1.394 (3)
N3—C27	1.360 (3)	C23—C24	1.460 (3)
C1—C2	1.378 (4)	C24—C25	1.390 (3)
C1—C5	1.474 (3)	C25—C26	1.417 (3)
C2—H2	0.9500	C25—C28	1.490 (3)
C2—C3	1.427 (3)	C26—H26	0.9500
C3—C4	1.389 (3)	C26—C27	1.386 (3)
C3—C12	1.474 (3)	C27—C35	1.466 (3)
C4—C19	1.473 (3)	C28—C29	1.387 (3)
C5—C6	1.382 (4)	C28—C33	1.393 (3)
C5—C10	1.399 (4)	C29—H29	0.9500
C6—H6	0.9500	C29—C30	1.387 (3)
C6—C7	1.391 (4)	C30—H30	0.9500
C7—H7	0.9500	C30—C31	1.382 (4)
C7—C8	1.384 (4)	C31—C32	1.391 (4)
C8—C9	1.372 (4)	C31—C34	1.510 (3)
C8—C11	1.511 (4)	C32—H32	0.9500
C9—H9	0.9500	C32—C33	1.394 (3)
C9—C10	1.393 (4)	C33—H33	0.9500
C10—H10	0.9500	C34—H34A	0.9800
C11—H11A	0.9800	C34—H34B	0.9800
C11—H11B	0.9800	C34—H34C	0.9800
C11—H11C	0.9800	C35—C36	1.395 (3)
C12—C13	1.392 (4)	C35—C40	1.395 (3)
C12—C17	1.400 (3)	C36—H36	0.9500
C13—H13	0.9500	C36—C37	1.388 (3)
C13—C14	1.391 (4)	C37—H37	0.9500
C14—H14	0.9500	C37—C38	1.396 (4)
C14—C15	1.391 (4)	C38—C39	1.386 (4)
C15—C16	1.388 (4)	C38—C41	1.513 (3)
C15—C18	1.510 (4)	C39—H39	0.9500
C16—H16	0.9500	C39—C40	1.388 (3)
C16—C17	1.391 (3)	C40—H40	0.9500
C17—H17	0.9500	C41—H41A	0.9800
C18—H18A	0.9800	C41—H41B	0.9800
C18—H18B	0.9800	C41—H41C	0.9800
C18—H18C	0.9800		
			
C1—N1—H1	129.5 (17)	C21—C20—C19	117.8 (2)
C1—N1—C4	111.0 (2)	C21—C20—H20	121.1
C4—N1—H1	119.0 (17)	C20—C21—H21	119.8
C19—N2—C23	119.21 (19)	C22—C21—C20	120.5 (2)
C24—N3—H3	117.5 (18)	C22—C21—H21	119.8
C27—N3—H3	130.8 (18)	C21—C22—H22	120.7
C27—N3—C24	111.56 (19)	C21—C22—C23	118.7 (2)
N1—C1—C2	106.8 (2)	C23—C22—H22	120.7
N1—C1—C5	120.2 (2)	N2—C23—C22	121.3 (2)
C2—C1—C5	132.9 (2)	N2—C23—C24	114.15 (19)
C1—C2—H2	125.8	C22—C23—C24	124.5 (2)
C1—C2—C3	108.5 (2)	N3—C24—C23	117.7 (2)
C3—C2—H2	125.8	N3—C24—C25	106.4 (2)
C2—C3—C12	124.5 (2)	C25—C24—C23	135.9 (2)
C4—C3—C2	106.6 (2)	C24—C25—C26	107.4 (2)
C4—C3—C12	128.7 (2)	C24—C25—C28	126.4 (2)
N1—C4—C3	107.1 (2)	C26—C25—C28	126.2 (2)
N1—C4—C19	116.7 (2)	C25—C26—H26	126.0
C3—C4—C19	136.2 (2)	C27—C26—C25	108.1 (2)
C6—C5—C1	121.3 (2)	C27—C26—H26	126.0
C6—C5—C10	117.9 (2)	N3—C27—C26	106.6 (2)
C10—C5—C1	120.8 (2)	N3—C27—C35	121.5 (2)
C5—C6—H6	119.5	C26—C27—C35	131.9 (2)
C5—C6—C7	121.0 (3)	C29—C28—C25	121.8 (2)
C7—C6—H6	119.5	C29—C28—C33	117.9 (2)
C6—C7—H7	119.3	C33—C28—C25	120.2 (2)
C8—C7—C6	121.3 (3)	C28—C29—H29	119.4
C8—C7—H7	119.3	C28—C29—C30	121.2 (2)
C7—C8—C11	121.3 (3)	C30—C29—H29	119.4
C9—C8—C7	117.6 (2)	C29—C30—H30	119.5
C9—C8—C11	121.2 (3)	C31—C30—C29	121.1 (2)
C8—C9—H9	119.0	C31—C30—H30	119.5
C8—C9—C10	122.0 (3)	C30—C31—C32	118.1 (2)
C10—C9—H9	119.0	C30—C31—C34	120.8 (2)
C5—C10—H10	119.9	C32—C31—C34	121.1 (2)
C9—C10—C5	120.1 (3)	C31—C32—H32	119.5
C9—C10—H10	119.9	C31—C32—C33	120.9 (2)
C8—C11—H11A	109.5	C33—C32—H32	119.5
C8—C11—H11B	109.5	C28—C33—C32	120.7 (2)
C8—C11—H11C	109.5	C28—C33—H33	119.6
H11A—C11—H11B	109.5	C32—C33—H33	119.6
H11A—C11—H11C	109.5	C31—C34—H34A	109.5
H11B—C11—H11C	109.5	C31—C34—H34B	109.5
C13—C12—C3	121.0 (2)	C31—C34—H34C	109.5
C13—C12—C17	117.7 (2)	H34A—C34—H34B	109.5
C17—C12—C3	121.3 (2)	H34A—C34—H34C	109.5
C12—C13—H13	119.3	H34B—C34—H34C	109.5
C14—C13—C12	121.4 (2)	C36—C35—C27	120.9 (2)
C14—C13—H13	119.3	C36—C35—C40	117.9 (2)
C13—C14—H14	119.6	C40—C35—C27	121.2 (2)
C13—C14—C15	120.9 (3)	C35—C36—H36	119.6
C15—C14—H14	119.6	C37—C36—C35	120.7 (2)
C14—C15—C18	121.4 (3)	C37—C36—H36	119.6
C16—C15—C14	118.0 (2)	C36—C37—H37	119.4
C16—C15—C18	120.5 (2)	C36—C37—C38	121.3 (2)
C15—C16—H16	119.3	C38—C37—H37	119.4
C15—C16—C17	121.4 (2)	C37—C38—C41	120.9 (2)
C17—C16—H16	119.3	C39—C38—C37	117.9 (2)
C12—C17—H17	119.6	C39—C38—C41	121.3 (2)
C16—C17—C12	120.7 (2)	C38—C39—H39	119.4
C16—C17—H17	119.6	C38—C39—C40	121.2 (2)
C15—C18—H18A	109.5	C40—C39—H39	119.4
C15—C18—H18B	109.5	C35—C40—H40	119.5
C15—C18—H18C	109.5	C39—C40—C35	121.1 (2)
H18A—C18—H18B	109.5	C39—C40—H40	119.5
H18A—C18—H18C	109.5	C38—C41—H41A	109.5
H18B—C18—H18C	109.5	C38—C41—H41B	109.5
N2—C19—C4	114.3 (2)	C38—C41—H41C	109.5
N2—C19—C20	122.3 (2)	H41A—C41—H41B	109.5
C20—C19—C4	123.3 (2)	H41A—C41—H41C	109.5
C19—C20—H20	121.1	H41B—C41—H41C	109.5
			
N1—C1—C2—C3	0.1 (3)	C15—C16—C17—C12	1.0 (4)
N1—C1—C5—C6	19.5 (4)	C17—C12—C13—C14	1.5 (4)
N1—C1—C5—C10	−160.0 (3)	C18—C15—C16—C17	178.2 (2)
N1—C4—C19—N2	−12.1 (3)	C19—N2—C23—C22	3.1 (3)
N1—C4—C19—C20	165.2 (2)	C19—N2—C23—C24	−175.5 (2)
N2—C19—C20—C21	−3.7 (4)	C19—C20—C21—C22	2.2 (4)
N2—C23—C24—N3	−3.0 (3)	C20—C21—C22—C23	1.7 (4)
N2—C23—C24—C25	176.4 (3)	C21—C22—C23—N2	−4.5 (3)
N3—C24—C25—C26	0.0 (3)	C21—C22—C23—C24	174.0 (2)
N3—C24—C25—C28	−178.2 (2)	C22—C23—C24—N3	178.4 (2)
N3—C27—C35—C36	−168.1 (2)	C22—C23—C24—C25	−2.2 (4)
N3—C27—C35—C40	11.4 (4)	C23—N2—C19—C4	178.4 (2)
C1—N1—C4—C3	1.4 (3)	C23—N2—C19—C20	1.0 (3)
C1—N1—C4—C19	−178.1 (2)	C23—C24—C25—C26	−179.5 (3)
C1—C2—C3—C4	0.8 (3)	C23—C24—C25—C28	2.3 (4)
C1—C2—C3—C12	−175.3 (2)	C24—N3—C27—C26	−0.3 (3)
C1—C5—C6—C7	−178.8 (3)	C24—N3—C27—C35	−179.6 (2)
C1—C5—C10—C9	179.1 (3)	C24—C25—C26—C27	−0.1 (3)
C2—C1—C5—C6	−156.7 (3)	C24—C25—C28—C29	−66.8 (3)
C2—C1—C5—C10	23.7 (5)	C24—C25—C28—C33	111.5 (3)
C2—C3—C4—N1	−1.3 (3)	C25—C26—C27—N3	0.3 (3)
C2—C3—C4—C19	178.1 (3)	C25—C26—C27—C35	179.5 (2)
C2—C3—C12—C13	−44.1 (4)	C25—C28—C29—C30	175.9 (2)
C2—C3—C12—C17	133.9 (3)	C25—C28—C33—C32	−176.0 (2)
C3—C4—C19—N2	168.6 (3)	C26—C25—C28—C29	115.4 (3)
C3—C4—C19—C20	−14.1 (4)	C26—C25—C28—C33	−66.3 (3)
C3—C12—C13—C14	179.6 (2)	C26—C27—C35—C36	12.8 (4)
C3—C12—C17—C16	−179.7 (2)	C26—C27—C35—C40	−167.8 (3)
C4—N1—C1—C2	−1.0 (3)	C27—N3—C24—C23	179.8 (2)
C4—N1—C1—C5	−178.1 (2)	C27—N3—C24—C25	0.2 (3)
C4—C3—C12—C13	140.8 (3)	C27—C35—C36—C37	−180.0 (2)
C4—C3—C12—C17	−41.2 (4)	C27—C35—C40—C39	−179.6 (2)
C4—C19—C20—C21	179.2 (2)	C28—C25—C26—C27	178.0 (2)
C5—C1—C2—C3	176.7 (3)	C28—C29—C30—C31	0.6 (4)
C5—C6—C7—C8	−0.4 (5)	C29—C28—C33—C32	2.4 (4)
C6—C5—C10—C9	−0.4 (5)	C29—C30—C31—C32	1.4 (4)
C6—C7—C8—C9	−0.4 (5)	C29—C30—C31—C34	−178.6 (2)
C6—C7—C8—C11	179.6 (3)	C30—C31—C32—C33	−1.5 (4)
C7—C8—C9—C10	0.7 (5)	C31—C32—C33—C28	−0.4 (4)
C8—C9—C10—C5	−0.3 (5)	C33—C28—C29—C30	−2.5 (4)
C10—C5—C6—C7	0.8 (5)	C34—C31—C32—C33	178.5 (2)
C11—C8—C9—C10	−179.2 (3)	C35—C36—C37—C38	−0.4 (4)
C12—C3—C4—N1	174.5 (2)	C36—C35—C40—C39	−0.1 (4)
C12—C3—C4—C19	−6.1 (5)	C36—C37—C38—C39	−0.2 (4)
C12—C13—C14—C15	−0.8 (4)	C36—C37—C38—C41	178.7 (2)
C13—C12—C17—C16	−1.6 (4)	C37—C38—C39—C40	0.6 (4)
C13—C14—C15—C16	0.2 (4)	C38—C39—C40—C35	−0.5 (4)
C13—C14—C15—C18	−178.3 (3)	C40—C35—C36—C37	0.6 (4)
C14—C15—C16—C17	−0.3 (4)	C41—C38—C39—C40	−178.3 (3)

### Carbonyl{2,2'-(pyridine-2,6-diyl)bis[3,5-bis(4-methylphenyl)pyrrolido-κN]}nickel(II) (pyrr2PyNiCO) Crystal data

**Table:**

[Ni(C_41_H_33_N_3_)(CO)]	*F*(000) = 684
*M**_r_* = 654.42	*D*_x_ = 1.342 Mg m^−^^3^
Monoclinic, *P*12_1_/*m*1	Mo *K*α radiation, λ = 0.71073 Å
*a* = 6.6482 (4) Å	Cell parameters from 5886 reflections
*b* = 27.1709 (18) Å	θ = 3.0–30.5°
*c* = 9.1322 (6) Å	µ = 0.64 mm^−^^1^
β = 101.0700 (12)°	*T* = 100 K
*V* = 1618.92 (18) Å^3^	Plates, yellow
*Z* = 2	0.36 × 0.27 × 0.05 mm

### Carbonyl{2,2'-(pyridine-2,6-diyl)bis[3,5-bis(4-methylphenyl)pyrrolido-κN]}nickel(II) (pyrr2PyNiCO) Data collection

**Table:**

Bruker D8 Quest with a Photon 100 CMOS detector diffractometer	4080 independent reflections
Radiation source: sealed tube	3280 reflections with *I* > 2σ(*I*)
Curved-graphite monochromator	*R*_int_ = 0.057
Detector resolution: 8 pixels mm^-1^	θ_max_ = 28.3°, θ_min_ = 3.0°
φ and ω scans	*h* = −8→8
Absorption correction: multi-scan (SADABS; Bruker, 2016)	*k* = −36→36
*T*_min_ = 0.859, *T*_max_ = 1.000	*l* = −12→12
18888 measured reflections	

### Carbonyl{2,2'-(pyridine-2,6-diyl)bis[3,5-bis(4-methylphenyl)pyrrolido-κN]}nickel(II) (pyrr2PyNiCO) Refinement

**Table:**

Refinement on *F*^2^	Primary atom site location: dual
Least-squares matrix: full	Secondary atom site location: difference Fourier map
*R*[*F*^2^ > 2σ(*F*^2^)] = 0.050	Hydrogen site location: inferred from neighbouring sites
*wR*(*F*^2^) = 0.119	H-atom parameters constrained
*S* = 1.07	*w* = 1/[σ^2^(*F*_o_^2^) + (0.0592*P*)^2^ + 0.9514*P*] where *P* = (*F*_o_^2^ + 2*F*_c_^2^)/3
4080 reflections	(Δ/σ)_max_ < 0.001
222 parameters	Δρ_max_ = 0.67 e Å^−^^3^
0 restraints	Δρ_min_ = −0.33 e Å^−^^3^

### Carbonyl{2,2'-(pyridine-2,6-diyl)bis[3,5-bis(4-methylphenyl)pyrrolido-κN]}nickel(II) (pyrr2PyNiCO) Special details

**Table:**

Geometry. All esds (except the esd in the dihedral angle between two l.s. planes) are estimated using the full covariance matrix. The cell esds are taken into account individually in the estimation of esds in distances, angles and torsion angles; correlations between esds in cell parameters are only used when they are defined by crystal symmetry. An approximate (isotropic) treatment of cell esds is used for estimating esds involving l.s. planes.

### Carbonyl{2,2'-(pyridine-2,6-diyl)bis[3,5-bis(4-methylphenyl)pyrrolido-κN]}nickel(II) (pyrr2PyNiCO) Fractional atomic coordinates and isotropic or equivalent isotropic displacement parameters (Å^2^)

**Table:**

	*x*	*y*	*z*	*U*_iso_*/*U*_eq_	
Ni	−0.04446 (6)	0.750000	0.86705 (4)	0.01505 (12)	
O	−0.2636 (4)	0.750000	0.5582 (3)	0.0313 (6)	
N1	0.1861 (4)	0.750000	1.0178 (3)	0.0161 (5)	
N2	−0.0255 (3)	0.81813 (7)	0.89233 (19)	0.0169 (4)	
C1	0.5450 (5)	0.750000	1.2176 (4)	0.0242 (7)	
H1	0.670105	0.750000	1.288274	0.029*	
C2	0.4567 (4)	0.79465 (9)	1.1677 (3)	0.0218 (5)	
H2	0.519973	0.824808	1.203580	0.026*	
C3	0.2721 (3)	0.79438 (8)	1.0632 (2)	0.0170 (4)	
C4	0.1516 (3)	0.83425 (8)	0.9875 (2)	0.0183 (5)	
C5	0.1610 (3)	0.88577 (8)	0.9845 (2)	0.0185 (5)	
C6	−0.0168 (3)	0.90088 (8)	0.8840 (2)	0.0205 (5)	
H6	−0.055029	0.933942	0.858799	0.025*	
C7	−0.1264 (3)	0.85917 (8)	0.8283 (2)	0.0179 (4)	
C8	0.3181 (3)	0.91905 (8)	1.0691 (2)	0.0185 (4)	
C9	0.5277 (4)	0.91026 (9)	1.0823 (3)	0.0221 (5)	
H9	0.572595	0.882978	1.031925	0.027*	
C10	0.6717 (4)	0.94084 (9)	1.1680 (3)	0.0229 (5)	
H10	0.813288	0.933585	1.176897	0.028*	
C11	0.6134 (4)	0.98169 (9)	1.2407 (3)	0.0221 (5)	
C12	0.4036 (4)	0.99210 (9)	1.2217 (3)	0.0233 (5)	
H12	0.359528	1.020579	1.267031	0.028*	
C13	0.2591 (4)	0.96129 (8)	1.1373 (3)	0.0218 (5)	
H13	0.117622	0.969085	1.125842	0.026*	
C14	0.7677 (4)	1.01476 (10)	1.3376 (3)	0.0294 (6)	
H14A	0.906304	1.002061	1.340151	0.044*	
H14B	0.739580	1.015472	1.439028	0.044*	
H14C	0.757029	1.048148	1.296201	0.044*	
C15	−0.3239 (3)	0.85618 (8)	0.7220 (2)	0.0174 (4)	
C16	−0.3557 (4)	0.88339 (8)	0.5890 (3)	0.0210 (5)	
H16	−0.250400	0.904361	0.568115	0.025*	
C17	−0.5400 (4)	0.87994 (9)	0.4874 (3)	0.0242 (5)	
H17	−0.558525	0.898537	0.397741	0.029*	
C18	−0.6980 (4)	0.84969 (10)	0.5148 (3)	0.0246 (5)	
C19	−0.6676 (4)	0.82337 (9)	0.6479 (3)	0.0243 (5)	
H19	−0.773984	0.802848	0.669221	0.029*	
C20	−0.4837 (4)	0.82661 (9)	0.7504 (3)	0.0218 (5)	
H20	−0.466766	0.808452	0.840810	0.026*	
C21	−0.8980 (4)	0.84547 (13)	0.4040 (3)	0.0388 (7)	
H21A	−0.898746	0.814872	0.347097	0.058*	
H21B	−0.912385	0.873591	0.335568	0.058*	
H21C	−1.012360	0.845229	0.457559	0.058*	
C22	−0.2023 (5)	0.750000	0.6821 (4)	0.0204 (7)	

### Carbonyl{2,2'-(pyridine-2,6-diyl)bis[3,5-bis(4-methylphenyl)pyrrolido-κN]}nickel(II) (pyrr2PyNiCO) Atomic displacement parameters (Å^2^)

**Table:**

	*U*^11^	*U*^22^	*U*^33^	*U*^12^	*U*^13^	*U*^23^
Ni	0.0181 (2)	0.0143 (2)	0.01106 (19)	0.000	−0.00126 (14)	0.000
O	0.0447 (16)	0.0315 (14)	0.0148 (12)	0.000	−0.0018 (11)	0.000
N1	0.0188 (13)	0.0167 (13)	0.0115 (12)	0.000	−0.0003 (10)	0.000
N2	0.0214 (10)	0.0150 (9)	0.0133 (9)	0.0003 (7)	0.0003 (7)	−0.0019 (7)
C1	0.0263 (17)	0.0288 (18)	0.0135 (15)	0.000	−0.0058 (13)	0.000
C2	0.0247 (12)	0.0216 (12)	0.0166 (11)	−0.0031 (9)	−0.0024 (9)	−0.0031 (9)
C3	0.0239 (11)	0.0160 (10)	0.0106 (9)	0.0006 (8)	0.0020 (8)	−0.0007 (8)
C4	0.0195 (11)	0.0190 (11)	0.0156 (10)	−0.0015 (9)	0.0012 (8)	−0.0008 (8)
C5	0.0216 (11)	0.0168 (11)	0.0169 (10)	−0.0008 (9)	0.0032 (9)	−0.0010 (8)
C6	0.0242 (12)	0.0150 (10)	0.0208 (11)	0.0002 (9)	0.0004 (9)	0.0004 (9)
C7	0.0203 (11)	0.0179 (11)	0.0145 (10)	0.0019 (9)	0.0009 (8)	0.0008 (8)
C8	0.0235 (11)	0.0158 (10)	0.0153 (10)	−0.0033 (9)	0.0012 (8)	0.0011 (8)
C9	0.0252 (12)	0.0207 (11)	0.0212 (11)	−0.0025 (9)	0.0066 (9)	−0.0023 (9)
C10	0.0181 (11)	0.0267 (12)	0.0245 (12)	−0.0038 (9)	0.0053 (9)	0.0000 (10)
C11	0.0236 (12)	0.0207 (11)	0.0209 (11)	−0.0062 (9)	0.0019 (9)	0.0033 (9)
C12	0.0263 (12)	0.0151 (11)	0.0273 (12)	−0.0009 (9)	0.0022 (10)	−0.0027 (9)
C13	0.0196 (11)	0.0164 (11)	0.0278 (12)	−0.0008 (9)	0.0001 (9)	0.0005 (9)
C14	0.0248 (13)	0.0307 (14)	0.0307 (13)	−0.0071 (10)	0.0009 (10)	−0.0061 (11)
C15	0.0198 (11)	0.0157 (10)	0.0157 (10)	0.0025 (8)	0.0007 (8)	−0.0027 (8)
C16	0.0256 (12)	0.0166 (11)	0.0207 (11)	0.0024 (9)	0.0039 (9)	0.0004 (9)
C17	0.0288 (13)	0.0256 (12)	0.0171 (11)	0.0091 (10)	0.0022 (9)	0.0021 (9)
C18	0.0214 (12)	0.0330 (14)	0.0185 (11)	0.0066 (10)	0.0017 (9)	−0.0026 (10)
C19	0.0213 (12)	0.0306 (13)	0.0215 (12)	−0.0017 (10)	0.0051 (9)	−0.0017 (10)
C20	0.0233 (12)	0.0236 (12)	0.0183 (11)	0.0022 (9)	0.0037 (9)	0.0012 (9)
C21	0.0231 (13)	0.067 (2)	0.0243 (13)	0.0050 (13)	0.0001 (10)	0.0025 (14)
C22	0.0260 (17)	0.0165 (15)	0.0185 (16)	0.000	0.0039 (13)	0.000

### Carbonyl{2,2'-(pyridine-2,6-diyl)bis[3,5-bis(4-methylphenyl)pyrrolido-κN]}nickel(II) (pyrr2PyNiCO) Geometric parameters (Å, º)

**Table:**

Ni—N1	1.853 (3)	C10—H10	0.9500
Ni—N2^i^	1.8667 (18)	C10—C11	1.386 (3)
Ni—N2	1.8666 (18)	C11—C12	1.401 (3)
Ni—C22	1.809 (3)	C11—C14	1.515 (3)
O—C22	1.126 (4)	C12—H12	0.9500
N1—C3^i^	1.364 (2)	C12—C13	1.391 (3)
N1—C3	1.364 (2)	C13—H13	0.9500
N2—C4	1.393 (3)	C14—H14A	0.9800
N2—C7	1.373 (3)	C14—H14B	0.9800
C1—H1	0.9500	C14—H14C	0.9800
C1—C2	1.386 (3)	C15—C16	1.402 (3)
C1—C2^i^	1.386 (3)	C15—C20	1.395 (3)
C2—H2	0.9500	C16—H16	0.9500
C2—C3	1.403 (3)	C16—C17	1.391 (3)
C3—C4	1.442 (3)	C17—H17	0.9500
C4—C5	1.402 (3)	C17—C18	1.394 (4)
C5—C6	1.411 (3)	C18—C19	1.391 (3)
C5—C8	1.482 (3)	C18—C21	1.512 (3)
C6—H6	0.9500	C19—H19	0.9500
C6—C7	1.390 (3)	C19—C20	1.392 (3)
C7—C15	1.478 (3)	C20—H20	0.9500
C8—C9	1.396 (3)	C21—H21A	0.9800
C8—C13	1.398 (3)	C21—H21B	0.9800
C9—H9	0.9500	C21—H21C	0.9800
C9—C10	1.390 (3)		
			
N1—Ni—N2	83.20 (6)	C11—C10—H10	119.2
N1—Ni—N2^i^	83.20 (6)	C10—C11—C12	117.7 (2)
N2—Ni—N2^i^	165.18 (11)	C10—C11—C14	122.3 (2)
C22—Ni—N1	160.41 (13)	C12—C11—C14	119.9 (2)
C22—Ni—N2	97.39 (5)	C11—C12—H12	119.6
C22—Ni—N2^i^	97.40 (6)	C13—C12—C11	120.8 (2)
C3^i^—N1—Ni	117.66 (13)	C13—C12—H12	119.6
C3—N1—Ni	117.66 (13)	C8—C13—H13	119.4
C3^i^—N1—C3	124.2 (3)	C12—C13—C8	121.2 (2)
C4—N2—Ni	114.70 (15)	C12—C13—H13	119.4
C7—N2—Ni	137.48 (15)	C11—C14—H14A	109.5
C7—N2—C4	107.19 (18)	C11—C14—H14B	109.5
C2—C1—H1	118.9	C11—C14—H14C	109.5
C2^i^—C1—H1	118.9	H14A—C14—H14B	109.5
C2^i^—C1—C2	122.2 (3)	H14A—C14—H14C	109.5
C1—C2—H2	120.7	H14B—C14—H14C	109.5
C1—C2—C3	118.6 (2)	C16—C15—C7	120.5 (2)
C3—C2—H2	120.7	C20—C15—C7	121.4 (2)
N1—C3—C2	118.2 (2)	C20—C15—C16	118.1 (2)
N1—C3—C4	110.88 (19)	C15—C16—H16	119.7
C2—C3—C4	130.9 (2)	C17—C16—C15	120.6 (2)
N2—C4—C3	112.82 (19)	C17—C16—H16	119.7
N2—C4—C5	109.72 (19)	C16—C17—H17	119.4
C5—C4—C3	137.4 (2)	C16—C17—C18	121.2 (2)
C4—C5—C6	105.6 (2)	C18—C17—H17	119.4
C4—C5—C8	129.0 (2)	C17—C18—C21	121.4 (2)
C6—C5—C8	125.5 (2)	C19—C18—C17	118.1 (2)
C5—C6—H6	125.8	C19—C18—C21	120.5 (2)
C7—C6—C5	108.4 (2)	C18—C19—H19	119.4
C7—C6—H6	125.8	C18—C19—C20	121.1 (2)
N2—C7—C6	109.10 (19)	C20—C19—H19	119.4
N2—C7—C15	122.3 (2)	C15—C20—H20	119.6
C6—C7—C15	128.5 (2)	C19—C20—C15	120.9 (2)
C9—C8—C5	122.2 (2)	C19—C20—H20	119.6
C9—C8—C13	117.6 (2)	C18—C21—H21A	109.5
C13—C8—C5	120.2 (2)	C18—C21—H21B	109.5
C8—C9—H9	119.5	C18—C21—H21C	109.5
C10—C9—C8	121.0 (2)	H21A—C21—H21B	109.5
C10—C9—H9	119.5	H21A—C21—H21C	109.5
C9—C10—H10	119.2	H21B—C21—H21C	109.5
C11—C10—C9	121.5 (2)	O—C22—Ni	166.1 (3)
			
Ni—N1—C3—C2	−174.47 (18)	C4—C5—C8—C9	45.6 (4)
Ni—N1—C3—C4	4.0 (3)	C4—C5—C8—C13	−135.3 (3)
Ni—N2—C4—C3	−7.8 (2)	C5—C6—C7—N2	1.1 (3)
Ni—N2—C4—C5	173.14 (15)	C5—C6—C7—C15	178.8 (2)
Ni—N2—C7—C6	−170.94 (18)	C5—C8—C9—C10	−177.0 (2)
Ni—N2—C7—C15	11.1 (4)	C5—C8—C13—C12	177.7 (2)
N1—Ni—N2—C4	7.95 (17)	C6—C5—C8—C9	−135.8 (2)
N1—Ni—N2—C7	177.3 (2)	C6—C5—C8—C13	43.2 (3)
N1—Ni—C22—O	0.000 (5)	C6—C7—C15—C16	48.5 (3)
N1—C3—C4—N2	2.5 (3)	C6—C7—C15—C20	−131.3 (3)
N1—C3—C4—C5	−178.9 (3)	C7—N2—C4—C3	179.68 (18)
N2^i^—Ni—N1—C3^i^	6.7 (2)	C7—N2—C4—C5	0.7 (3)
N2^i^—Ni—N1—C3	179.2 (2)	C7—C15—C16—C17	178.8 (2)
N2—Ni—N1—C3	−6.7 (2)	C7—C15—C20—C19	−178.7 (2)
N2—Ni—N1—C3^i^	−179.2 (2)	C8—C5—C6—C7	−179.5 (2)
N2^i^—Ni—N2—C4	31.5 (6)	C8—C9—C10—C11	−1.4 (4)
N2^i^—Ni—N2—C7	−159.1 (3)	C9—C8—C13—C12	−3.2 (3)
N2—Ni—C22—O	90.49 (6)	C9—C10—C11—C12	−1.8 (4)
N2^i^—Ni—C22—O	−90.49 (6)	C9—C10—C11—C14	178.5 (2)
N2—C4—C5—C6	0.0 (3)	C10—C11—C12—C13	2.4 (4)
N2—C4—C5—C8	178.8 (2)	C11—C12—C13—C8	0.1 (4)
N2—C7—C15—C16	−134.0 (2)	C13—C8—C9—C10	3.9 (3)
N2—C7—C15—C20	46.2 (3)	C14—C11—C12—C13	−177.8 (2)
C1—C2—C3—N1	1.2 (4)	C15—C16—C17—C18	0.3 (4)
C1—C2—C3—C4	−176.9 (3)	C16—C15—C20—C19	1.5 (3)
C2^i^—C1—C2—C3	−0.1 (5)	C16—C17—C18—C19	0.8 (4)
C2—C3—C4—N2	−179.3 (2)	C16—C17—C18—C21	−179.4 (2)
C2—C3—C4—C5	−0.6 (5)	C17—C18—C19—C20	−0.8 (4)
C3^i^—N1—C3—C2	−2.6 (4)	C18—C19—C20—C15	−0.4 (4)
C3^i^—N1—C3—C4	175.9 (2)	C20—C15—C16—C17	−1.4 (3)
C3—C4—C5—C6	−178.7 (3)	C21—C18—C19—C20	179.5 (2)
C3—C4—C5—C8	0.1 (5)	C22—Ni—N1—C3^i^	−86.2 (2)
C4—N2—C7—C6	−1.1 (3)	C22—Ni—N1—C3	86.2 (2)
C4—N2—C7—C15	−179.0 (2)	C22—Ni—N2—C4	−152.32 (18)
C4—C5—C6—C7	−0.7 (3)	C22—Ni—N2—C7	17.0 (3)

Symmetry code: (i) *x*, −*y*+3/2, *z*.

### Ammine{2,2'-(pyridine-2,6-diyl)bis[3,5-bis(4-methylphenyl)pyrrolido-κN]}nickel(II) (pyrr2PyNiNH3) Crystal data

**Table:**

[Ni(C_41_H_33_N_3_)(NH_3_)]	*F*(000) = 1352
*M**_r_* = 643.45	*D*_x_ = 1.313 Mg m^−^^3^
Monoclinic, *P*2_1_/*c*	Mo *K*α radiation, λ = 0.71073 Å
*a* = 15.9773 (6) Å	Cell parameters from 9841 reflections
*b* = 14.9441 (5) Å	θ = 3.0–39.4°
*c* = 14.3238 (5) Å	µ = 0.63 mm^−^^1^
β = 107.8140 (8)°	*T* = 100 K
*V* = 3256.1 (2) Å^3^	Plates, red
*Z* = 4	0.46 × 0.41 × 0.14 mm

### Ammine{2,2'-(pyridine-2,6-diyl)bis[3,5-bis(4-methylphenyl)pyrrolido-κN]}nickel(II) (pyrr2PyNiNH3) Data collection

**Table:**

Bruker D8 Quest with a Photon 100 CMOS detector diffractometer	8502 reflections with *I* > 2σ(*I*)
Curved-graphite monochromator	*R*_int_ = 0.026
φ and ω scans	θ_max_ = 30.5°, θ_min_ = 2.9°
Absorption correction: multi-scan (SADABS; Bruker, 2016)	*h* = −22→22
*T*_min_ = 0.858, *T*_max_ = 0.967	*k* = −21→21
48278 measured reflections	*l* = −20→20
9931 independent reflections	

### Ammine{2,2'-(pyridine-2,6-diyl)bis[3,5-bis(4-methylphenyl)pyrrolido-κN]}nickel(II) (pyrr2PyNiNH3) Refinement

**Table:**

Refinement on *F*^2^	Primary atom site location: dual
Least-squares matrix: full	Secondary atom site location: difference Fourier map
*R*[*F*^2^ > 2σ(*F*^2^)] = 0.036	Hydrogen site location: mixed
*wR*(*F*^2^) = 0.098	H atoms treated by a mixture of independent and constrained refinement
*S* = 1.05	*w* = 1/[σ^2^(*F*_o_^2^) + (0.0562*P*)^2^ + 1.1192*P*] where *P* = (*F*_o_^2^ + 2*F*_c_^2^)/3
9931 reflections	(Δ/σ)_max_ = 0.002
431 parameters	Δρ_max_ = 0.51 e Å^−^^3^
0 restraints	Δρ_min_ = −0.25 e Å^−^^3^

### Ammine{2,2'-(pyridine-2,6-diyl)bis[3,5-bis(4-methylphenyl)pyrrolido-κN]}nickel(II) (pyrr2PyNiNH3) Special details

**Table:**

Geometry. All esds (except the esd in the dihedral angle between two l.s. planes) are estimated using the full covariance matrix. The cell esds are taken into account individually in the estimation of esds in distances, angles and torsion angles; correlations between esds in cell parameters are only used when they are defined by crystal symmetry. An approximate (isotropic) treatment of cell esds is used for estimating esds involving l.s. planes.

### Ammine{2,2'-(pyridine-2,6-diyl)bis[3,5-bis(4-methylphenyl)pyrrolido-κN]}nickel(II) (pyrr2PyNiNH3) Fractional atomic coordinates and isotropic or equivalent isotropic displacement parameters (Å^2^)

**Table:**

	*x*	*y*	*z*	*U*_iso_*/*U*_eq_	
Ni	0.10799 (2)	0.41253 (2)	0.93990 (2)	0.01428 (5)	
N1	0.21118 (7)	0.43570 (7)	0.90627 (7)	0.01684 (19)	
N2	0.05856 (7)	0.39854 (7)	0.80601 (7)	0.01515 (19)	
N3	−0.00188 (7)	0.36827 (7)	0.94266 (7)	0.01631 (19)	
N4	0.14688 (8)	0.46458 (8)	1.06929 (8)	0.0179 (2)	
H4A	0.1037 (17)	0.4707 (17)	1.0960 (18)	0.060 (7)*	
H4B	0.1871 (18)	0.4399 (19)	1.1142 (19)	0.063 (7)*	
H4C	0.1692 (16)	0.5156 (18)	1.0630 (17)	0.056 (7)*	
C1	0.29896 (8)	0.45061 (9)	0.94997 (9)	0.0190 (2)	
C2	0.33888 (8)	0.47073 (10)	0.87809 (10)	0.0227 (3)	
H2	0.399240	0.484362	0.889319	0.027*	
C3	0.27366 (8)	0.46719 (9)	0.78618 (9)	0.0188 (2)	
C4	0.19592 (8)	0.44495 (9)	0.80609 (9)	0.0171 (2)	
C5	0.10722 (8)	0.42434 (8)	0.74805 (9)	0.0162 (2)	
C6	0.06831 (9)	0.42720 (9)	0.64636 (9)	0.0185 (2)	
H6	0.101605	0.443252	0.604162	0.022*	
C7	−0.02032 (9)	0.40596 (9)	0.60869 (9)	0.0200 (2)	
H7	−0.047903	0.408038	0.539816	0.024*	
C8	−0.06959 (8)	0.38168 (9)	0.66984 (9)	0.0190 (2)	
H8	−0.130417	0.368353	0.643358	0.023*	
C9	−0.02792 (8)	0.37728 (8)	0.77075 (9)	0.0159 (2)	
C10	−0.06230 (8)	0.35450 (8)	0.85048 (9)	0.0162 (2)	
C11	−0.14162 (8)	0.32195 (8)	0.86028 (9)	0.0170 (2)	
C12	−0.12798 (8)	0.31616 (9)	0.96247 (9)	0.0186 (2)	
H12	−0.169888	0.296952	0.993071	0.022*	
C13	−0.04184 (8)	0.34373 (8)	1.01047 (9)	0.0172 (2)	
C14	0.34369 (8)	0.43631 (9)	1.05496 (9)	0.0196 (2)	
C15	0.32402 (9)	0.36205 (10)	1.10428 (10)	0.0225 (3)	
H15	0.278667	0.322055	1.070416	0.027*	
C16	0.37015 (10)	0.34625 (10)	1.20222 (10)	0.0264 (3)	
H16	0.354631	0.296548	1.234851	0.032*	
C17	0.43869 (9)	0.40205 (11)	1.25325 (10)	0.0273 (3)	
C18	0.45818 (9)	0.47588 (11)	1.20459 (11)	0.0274 (3)	
H18	0.504218	0.515165	1.238430	0.033*	
C19	0.41132 (9)	0.49320 (10)	1.10702 (10)	0.0233 (3)	
H19	0.425507	0.544340	1.075466	0.028*	
C20	0.49213 (12)	0.38050 (14)	1.35736 (12)	0.0393 (4)	
H20A	0.458982	0.339379	1.386063	0.059*	
H20B	0.504500	0.435775	1.395978	0.059*	
H20C	0.547612	0.352361	1.357653	0.059*	
C21	0.28514 (8)	0.48099 (9)	0.68838 (9)	0.0186 (2)	
C22	0.32480 (11)	0.41602 (10)	0.64649 (11)	0.0266 (3)	
H22	0.346065	0.362588	0.681707	0.032*	
C23	0.33372 (12)	0.42831 (11)	0.55354 (11)	0.0308 (3)	
H23	0.360985	0.383006	0.526433	0.037*	
C24	0.30348 (10)	0.50564 (10)	0.49969 (10)	0.0245 (3)	
C25	0.26657 (10)	0.57145 (10)	0.54301 (10)	0.0235 (3)	
H25	0.247296	0.625774	0.508725	0.028*	
C26	0.25718 (9)	0.55955 (10)	0.63571 (10)	0.0231 (3)	
H26	0.231342	0.605643	0.663410	0.028*	
C27	0.31087 (14)	0.51780 (13)	0.39833 (12)	0.0414 (4)	
H27A	0.362859	0.485913	0.393138	0.062*	
H27B	0.316402	0.581651	0.385761	0.062*	
H27C	0.258167	0.493772	0.350014	0.062*	
C28	0.00284 (8)	0.34193 (8)	1.11671 (9)	0.0179 (2)	
C29	−0.03979 (9)	0.37551 (9)	1.18122 (10)	0.0215 (2)	
H29	−0.096129	0.402330	1.155903	0.026*	
C30	−0.00052 (11)	0.37002 (9)	1.28201 (10)	0.0257 (3)	
H30	−0.031062	0.391957	1.324719	0.031*	
C31	0.08258 (10)	0.33305 (9)	1.32141 (10)	0.0254 (3)	
C32	0.12548 (10)	0.29963 (9)	1.25727 (10)	0.0238 (3)	
H32	0.182468	0.274300	1.282871	0.029*	
C33	0.08576 (9)	0.30297 (9)	1.15627 (9)	0.0202 (2)	
H33	0.115269	0.278520	1.113748	0.024*	
C34	0.12424 (13)	0.32882 (12)	1.43072 (11)	0.0371 (4)	
H34A	0.148744	0.387517	1.454991	0.056*	
H34B	0.171345	0.284084	1.446531	0.056*	
H34C	0.079770	0.312095	1.461879	0.056*	
C35	−0.22326 (8)	0.29502 (8)	0.78474 (9)	0.0181 (2)	
C36	−0.22213 (8)	0.24553 (9)	0.70235 (10)	0.0205 (2)	
H36	−0.167373	0.230297	0.693374	0.025*	
C37	−0.29980 (9)	0.21818 (10)	0.63324 (10)	0.0235 (3)	
H37	−0.297210	0.184450	0.577976	0.028*	
C38	−0.38148 (9)	0.23948 (10)	0.64380 (11)	0.0250 (3)	
C39	−0.38268 (9)	0.28783 (10)	0.72610 (11)	0.0263 (3)	
H39	−0.437551	0.302475	0.735130	0.032*	
C40	−0.30516 (9)	0.31540 (10)	0.79584 (10)	0.0232 (3)	
H40	−0.307994	0.348378	0.851496	0.028*	
C41	−0.46521 (10)	0.20943 (12)	0.56827 (12)	0.0343 (3)	
H41A	−0.480968	0.149536	0.585162	0.051*	
H41B	−0.456395	0.207820	0.503525	0.051*	
H41C	−0.512642	0.251416	0.567071	0.051*	

### Ammine{2,2'-(pyridine-2,6-diyl)bis[3,5-bis(4-methylphenyl)pyrrolido-κN]}nickel(II) (pyrr2PyNiNH3) Atomic displacement parameters (Å^2^)

**Table:**

	*U*^11^	*U*^22^	*U*^33^	*U*^12^	*U*^13^	*U*^23^
Ni	0.01402 (8)	0.01717 (8)	0.01271 (8)	−0.00050 (5)	0.00565 (6)	0.00038 (5)
N1	0.0149 (5)	0.0218 (5)	0.0145 (4)	−0.0004 (4)	0.0055 (4)	0.0009 (4)
N2	0.0152 (4)	0.0168 (5)	0.0146 (4)	0.0003 (4)	0.0063 (4)	0.0006 (3)
N3	0.0164 (5)	0.0190 (5)	0.0145 (4)	−0.0014 (4)	0.0062 (4)	0.0005 (4)
N4	0.0192 (5)	0.0189 (5)	0.0165 (5)	−0.0006 (4)	0.0070 (4)	−0.0003 (4)
C1	0.0156 (5)	0.0250 (6)	0.0168 (5)	−0.0010 (4)	0.0055 (4)	0.0006 (5)
C2	0.0157 (5)	0.0339 (7)	0.0198 (6)	−0.0026 (5)	0.0074 (5)	0.0014 (5)
C3	0.0178 (5)	0.0237 (6)	0.0167 (5)	−0.0007 (4)	0.0079 (4)	0.0011 (4)
C4	0.0168 (5)	0.0205 (6)	0.0151 (5)	−0.0002 (4)	0.0064 (4)	0.0011 (4)
C5	0.0169 (5)	0.0171 (5)	0.0161 (5)	0.0006 (4)	0.0071 (4)	0.0001 (4)
C6	0.0214 (6)	0.0206 (6)	0.0151 (5)	−0.0011 (4)	0.0080 (4)	−0.0001 (4)
C7	0.0216 (6)	0.0235 (6)	0.0144 (5)	−0.0015 (5)	0.0049 (5)	−0.0003 (4)
C8	0.0172 (5)	0.0227 (6)	0.0166 (5)	−0.0015 (4)	0.0045 (4)	−0.0003 (4)
C9	0.0161 (5)	0.0158 (5)	0.0167 (5)	0.0002 (4)	0.0065 (4)	−0.0004 (4)
C10	0.0161 (5)	0.0179 (5)	0.0154 (5)	−0.0003 (4)	0.0061 (4)	−0.0003 (4)
C11	0.0165 (5)	0.0162 (5)	0.0195 (5)	−0.0009 (4)	0.0072 (4)	−0.0010 (4)
C12	0.0196 (6)	0.0191 (6)	0.0198 (5)	−0.0020 (4)	0.0099 (5)	0.0007 (4)
C13	0.0202 (5)	0.0169 (5)	0.0168 (5)	−0.0004 (4)	0.0089 (4)	0.0006 (4)
C14	0.0145 (5)	0.0274 (6)	0.0175 (5)	0.0028 (5)	0.0057 (4)	0.0004 (5)
C15	0.0199 (6)	0.0261 (7)	0.0210 (6)	0.0009 (5)	0.0056 (5)	0.0010 (5)
C16	0.0259 (7)	0.0308 (7)	0.0224 (6)	0.0046 (5)	0.0074 (5)	0.0052 (5)
C17	0.0211 (6)	0.0423 (9)	0.0177 (6)	0.0060 (6)	0.0047 (5)	−0.0007 (5)
C18	0.0181 (6)	0.0398 (8)	0.0230 (6)	−0.0020 (5)	0.0044 (5)	−0.0058 (6)
C19	0.0179 (6)	0.0303 (7)	0.0222 (6)	−0.0013 (5)	0.0071 (5)	−0.0014 (5)
C20	0.0319 (8)	0.0580 (11)	0.0229 (7)	0.0069 (8)	0.0007 (6)	0.0023 (7)
C21	0.0162 (5)	0.0238 (6)	0.0173 (5)	−0.0025 (4)	0.0075 (4)	0.0002 (4)
C22	0.0366 (8)	0.0233 (7)	0.0228 (6)	0.0056 (5)	0.0137 (6)	0.0039 (5)
C23	0.0443 (9)	0.0289 (7)	0.0246 (7)	0.0100 (6)	0.0186 (6)	0.0018 (5)
C24	0.0297 (7)	0.0277 (7)	0.0194 (6)	0.0019 (5)	0.0122 (5)	0.0017 (5)
C25	0.0269 (7)	0.0246 (6)	0.0199 (6)	0.0028 (5)	0.0082 (5)	0.0038 (5)
C26	0.0253 (6)	0.0255 (6)	0.0211 (6)	0.0049 (5)	0.0107 (5)	0.0012 (5)
C27	0.0639 (12)	0.0437 (10)	0.0251 (7)	0.0160 (9)	0.0263 (8)	0.0079 (7)
C28	0.0229 (6)	0.0163 (5)	0.0165 (5)	−0.0031 (4)	0.0089 (5)	0.0011 (4)
C29	0.0277 (6)	0.0182 (6)	0.0220 (6)	−0.0020 (5)	0.0126 (5)	0.0005 (5)
C30	0.0409 (8)	0.0204 (6)	0.0208 (6)	−0.0034 (5)	0.0169 (6)	−0.0027 (5)
C31	0.0396 (8)	0.0198 (6)	0.0168 (6)	−0.0074 (5)	0.0085 (5)	0.0002 (5)
C32	0.0269 (6)	0.0232 (6)	0.0193 (6)	−0.0029 (5)	0.0042 (5)	0.0026 (5)
C33	0.0246 (6)	0.0194 (6)	0.0182 (5)	−0.0022 (5)	0.0091 (5)	0.0015 (4)
C34	0.0552 (10)	0.0357 (8)	0.0172 (6)	−0.0063 (7)	0.0062 (7)	0.0004 (6)
C35	0.0162 (5)	0.0183 (6)	0.0210 (6)	−0.0011 (4)	0.0074 (4)	0.0016 (4)
C36	0.0172 (5)	0.0216 (6)	0.0239 (6)	−0.0020 (4)	0.0080 (5)	−0.0016 (5)
C37	0.0227 (6)	0.0250 (6)	0.0228 (6)	−0.0056 (5)	0.0070 (5)	−0.0015 (5)
C38	0.0183 (6)	0.0282 (7)	0.0270 (6)	−0.0067 (5)	0.0049 (5)	0.0054 (5)
C39	0.0160 (6)	0.0327 (7)	0.0319 (7)	−0.0012 (5)	0.0099 (5)	0.0030 (6)
C40	0.0192 (6)	0.0274 (7)	0.0264 (6)	−0.0011 (5)	0.0120 (5)	−0.0003 (5)
C41	0.0215 (7)	0.0433 (9)	0.0332 (8)	−0.0101 (6)	0.0011 (6)	0.0045 (7)

### Ammine{2,2'-(pyridine-2,6-diyl)bis[3,5-bis(4-methylphenyl)pyrrolido-κN]}nickel(II) (pyrr2PyNiNH3) Geometric parameters (Å, º)

**Table:**

Ni—N1	1.8858 (10)	C20—H20A	0.9800
Ni—N2	1.8490 (10)	C20—H20B	0.9800
Ni—N3	1.8876 (10)	C20—H20C	0.9800
Ni—N4	1.9291 (11)	C21—C22	1.3917 (19)
N1—C1	1.3679 (16)	C21—C26	1.3932 (19)
N1—C4	1.3871 (15)	C22—H22	0.9500
N2—C5	1.3554 (15)	C22—C23	1.394 (2)
N2—C9	1.3558 (16)	C23—H23	0.9500
N3—C10	1.3920 (15)	C23—C24	1.391 (2)
N3—C13	1.3655 (15)	C24—C25	1.3871 (19)
N4—H4A	0.89 (3)	C24—C27	1.5033 (19)
N4—H4B	0.84 (3)	C25—H25	0.9500
N4—H4C	0.86 (3)	C25—C26	1.3925 (18)
C1—C2	1.3996 (17)	C26—H26	0.9500
C1—C14	1.4699 (17)	C27—H27A	0.9800
C2—H2	0.9500	C27—H27B	0.9800
C2—C3	1.4080 (18)	C27—H27C	0.9800
C3—C4	1.3968 (17)	C28—C29	1.3981 (17)
C3—C21	1.4818 (17)	C28—C33	1.3986 (19)
C4—C5	1.4400 (17)	C29—H29	0.9500
C5—C6	1.3986 (17)	C29—C30	1.3894 (19)
C6—H6	0.9500	C30—H30	0.9500
C6—C7	1.3891 (18)	C30—C31	1.389 (2)
C7—H7	0.9500	C31—C32	1.396 (2)
C7—C8	1.3930 (18)	C31—C34	1.5036 (19)
C8—H8	0.9500	C32—H32	0.9500
C8—C9	1.3957 (17)	C32—C33	1.3915 (18)
C9—C10	1.4511 (16)	C33—H33	0.9500
C10—C11	1.4040 (16)	C34—H34A	0.9800
C11—C12	1.4151 (17)	C34—H34B	0.9800
C11—C35	1.4733 (17)	C34—H34C	0.9800
C12—H12	0.9500	C35—C36	1.3976 (18)
C12—C13	1.3992 (17)	C35—C40	1.3993 (17)
C13—C28	1.4702 (17)	C36—H36	0.9500
C14—C15	1.4020 (19)	C36—C37	1.3912 (18)
C14—C19	1.3976 (19)	C37—H37	0.9500
C15—H15	0.9500	C37—C38	1.396 (2)
C15—C16	1.3907 (19)	C38—C39	1.388 (2)
C16—H16	0.9500	C38—C41	1.509 (2)
C16—C17	1.393 (2)	C39—H39	0.9500
C17—C18	1.390 (2)	C39—C40	1.3945 (19)
C17—C20	1.509 (2)	C40—H40	0.9500
C18—H18	0.9500	C41—H41A	0.9800
C18—C19	1.3929 (19)	C41—H41B	0.9800
C19—H19	0.9500	C41—H41C	0.9800
			
N1—Ni—N3	163.95 (5)	C17—C20—H20A	109.5
N1—Ni—N4	96.98 (5)	C17—C20—H20B	109.5
N2—Ni—N1	83.41 (4)	C17—C20—H20C	109.5
N2—Ni—N3	82.95 (4)	H20A—C20—H20B	109.5
N2—Ni—N4	162.16 (5)	H20A—C20—H20C	109.5
N3—Ni—N4	98.59 (5)	H20B—C20—H20C	109.5
C1—N1—Ni	140.10 (9)	C22—C21—C3	120.99 (12)
C1—N1—C4	106.75 (10)	C22—C21—C26	117.86 (12)
C4—N1—Ni	113.07 (8)	C26—C21—C3	121.15 (12)
C5—N2—Ni	117.40 (8)	C21—C22—H22	119.6
C5—N2—C9	123.28 (11)	C21—C22—C23	120.86 (13)
C9—N2—Ni	118.43 (8)	C23—C22—H22	119.6
C10—N3—Ni	114.29 (8)	C22—C23—H23	119.4
C13—N3—Ni	138.51 (9)	C24—C23—C22	121.30 (13)
C13—N3—C10	107.19 (10)	C24—C23—H23	119.4
Ni—N4—H4A	113.0 (16)	C23—C24—C27	121.29 (13)
Ni—N4—H4B	120.3 (18)	C25—C24—C23	117.64 (12)
Ni—N4—H4C	106.2 (16)	C25—C24—C27	121.07 (13)
H4A—N4—H4B	103 (2)	C24—C25—H25	119.3
H4A—N4—H4C	111 (2)	C24—C25—C26	121.37 (13)
H4B—N4—H4C	103 (2)	C26—C25—H25	119.3
N1—C1—C2	109.43 (11)	C21—C26—H26	119.5
N1—C1—C14	123.64 (11)	C25—C26—C21	120.91 (13)
C2—C1—C14	126.55 (11)	C25—C26—H26	119.5
C1—C2—H2	126.1	C24—C27—H27A	109.5
C1—C2—C3	107.83 (11)	C24—C27—H27B	109.5
C3—C2—H2	126.1	C24—C27—H27C	109.5
C2—C3—C21	127.47 (11)	H27A—C27—H27B	109.5
C4—C3—C2	105.55 (11)	H27A—C27—H27C	109.5
C4—C3—C21	126.95 (11)	H27B—C27—H27C	109.5
N1—C4—C3	110.43 (11)	C29—C28—C13	119.81 (12)
N1—C4—C5	113.98 (10)	C29—C28—C33	118.31 (12)
C3—C4—C5	135.45 (11)	C33—C28—C13	121.79 (11)
N2—C5—C4	110.79 (11)	C28—C29—H29	119.7
N2—C5—C6	119.41 (11)	C30—C29—C28	120.60 (13)
C6—C5—C4	129.80 (11)	C30—C29—H29	119.7
C5—C6—H6	120.9	C29—C30—H30	119.4
C7—C6—C5	118.23 (11)	C31—C30—C29	121.17 (13)
C7—C6—H6	120.9	C31—C30—H30	119.4
C6—C7—H7	119.3	C30—C31—C32	118.41 (12)
C6—C7—C8	121.36 (12)	C30—C31—C34	120.24 (14)
C8—C7—H7	119.3	C32—C31—C34	121.35 (15)
C7—C8—H8	120.6	C31—C32—H32	119.6
C7—C8—C9	118.76 (12)	C33—C32—C31	120.78 (13)
C9—C8—H8	120.6	C33—C32—H32	119.6
N2—C9—C8	118.92 (11)	C28—C33—H33	119.7
N2—C9—C10	110.48 (10)	C32—C33—C28	120.69 (12)
C8—C9—C10	130.58 (11)	C32—C33—H33	119.7
N3—C10—C9	113.09 (10)	C31—C34—H34A	109.5
N3—C10—C11	109.98 (10)	C31—C34—H34B	109.5
C11—C10—C9	136.93 (11)	C31—C34—H34C	109.5
C10—C11—C12	105.46 (11)	H34A—C34—H34B	109.5
C10—C11—C35	130.14 (11)	H34A—C34—H34C	109.5
C12—C11—C35	124.37 (11)	H34B—C34—H34C	109.5
C11—C12—H12	126.1	C36—C35—C11	121.84 (11)
C13—C12—C11	107.90 (11)	C36—C35—C40	117.73 (12)
C13—C12—H12	126.1	C40—C35—C11	120.38 (12)
N3—C13—C12	109.47 (11)	C35—C36—H36	119.4
N3—C13—C28	123.82 (11)	C37—C36—C35	121.14 (12)
C12—C13—C28	126.61 (11)	C37—C36—H36	119.4
C15—C14—C1	121.12 (12)	C36—C37—H37	119.5
C19—C14—C1	120.80 (12)	C36—C37—C38	121.07 (13)
C19—C14—C15	117.94 (12)	C38—C37—H37	119.5
C14—C15—H15	119.6	C37—C38—C41	120.51 (14)
C16—C15—C14	120.76 (13)	C39—C38—C37	117.85 (13)
C16—C15—H15	119.6	C39—C38—C41	121.63 (14)
C15—C16—H16	119.4	C38—C39—H39	119.3
C15—C16—C17	121.11 (14)	C38—C39—C40	121.48 (13)
C17—C16—H16	119.4	C40—C39—H39	119.3
C16—C17—C20	120.48 (15)	C35—C40—H40	119.6
C18—C17—C16	118.22 (13)	C39—C40—C35	120.72 (13)
C18—C17—C20	121.26 (15)	C39—C40—H40	119.6
C17—C18—H18	119.5	C38—C41—H41A	109.5
C17—C18—C19	121.09 (14)	C38—C41—H41B	109.5
C19—C18—H18	119.5	C38—C41—H41C	109.5
C14—C19—H19	119.6	H41A—C41—H41B	109.5
C18—C19—C14	120.85 (14)	H41A—C41—H41C	109.5
C18—C19—H19	119.6	H41B—C41—H41C	109.5
			
Ni—N1—C1—C2	−175.37 (11)	C5—N2—C9—C10	−178.59 (11)
Ni—N1—C1—C14	11.3 (2)	C5—C6—C7—C8	−0.50 (19)
Ni—N1—C4—C3	176.50 (9)	C6—C7—C8—C9	−1.2 (2)
Ni—N1—C4—C5	−7.17 (14)	C7—C8—C9—N2	1.34 (18)
Ni—N2—C5—C4	9.41 (13)	C7—C8—C9—C10	179.84 (13)
Ni—N2—C5—C6	−170.85 (9)	C8—C9—C10—N3	−172.66 (13)
Ni—N2—C9—C8	169.03 (9)	C8—C9—C10—C11	7.1 (3)
Ni—N2—C9—C10	−9.75 (14)	C9—N2—C5—C4	178.36 (11)
Ni—N3—C10—C9	−0.02 (13)	C9—N2—C5—C6	−1.90 (18)
Ni—N3—C10—C11	−179.83 (8)	C9—C10—C11—C12	−179.68 (14)
Ni—N3—C13—C12	179.52 (10)	C9—C10—C11—C35	2.3 (2)
Ni—N3—C13—C28	−4.1 (2)	C10—N3—C13—C12	−1.25 (14)
N1—Ni—N2—C5	−10.93 (9)	C10—N3—C13—C28	175.14 (11)
N1—Ni—N2—C9	179.57 (10)	C10—C11—C12—C13	−0.82 (14)
N1—Ni—N3—C10	−36.0 (2)	C10—C11—C35—C36	41.5 (2)
N1—Ni—N3—C13	143.17 (16)	C10—C11—C35—C40	−141.21 (14)
N1—C1—C2—C3	−0.70 (16)	C11—C12—C13—N3	1.30 (15)
N1—C1—C14—C15	40.6 (2)	C11—C12—C13—C28	−174.95 (12)
N1—C1—C14—C19	−143.73 (13)	C11—C35—C36—C37	177.93 (12)
N1—C4—C5—N2	−1.13 (15)	C11—C35—C40—C39	−178.08 (13)
N1—C4—C5—C6	179.17 (12)	C12—C11—C35—C36	−136.15 (14)
N2—Ni—N1—C1	−174.10 (15)	C12—C11—C35—C40	41.12 (19)
N2—Ni—N1—C4	9.65 (9)	C12—C13—C28—C29	−47.47 (19)
N2—Ni—N3—C10	−4.08 (9)	C12—C13—C28—C33	129.04 (14)
N2—Ni—N3—C13	175.12 (14)	C13—N3—C10—C9	−179.46 (10)
N2—C5—C6—C7	2.00 (18)	C13—N3—C10—C11	0.73 (14)
N2—C9—C10—N3	5.93 (15)	C13—C28—C29—C30	176.54 (12)
N2—C9—C10—C11	−174.33 (14)	C13—C28—C33—C32	−177.98 (12)
N3—Ni—N1—C1	−142.19 (16)	C14—C1—C2—C3	172.34 (13)
N3—Ni—N1—C4	41.6 (2)	C14—C15—C16—C17	−1.9 (2)
N3—Ni—N2—C5	177.54 (10)	C15—C14—C19—C18	0.7 (2)
N3—Ni—N2—C9	8.03 (9)	C15—C16—C17—C18	2.0 (2)
N3—C10—C11—C12	0.07 (14)	C15—C16—C17—C20	−175.81 (14)
N3—C10—C11—C35	−177.94 (12)	C16—C17—C18—C19	−0.8 (2)
N3—C13—C28—C29	136.78 (13)	C17—C18—C19—C14	−0.5 (2)
N3—C13—C28—C33	−46.72 (18)	C19—C14—C15—C16	0.5 (2)
N4—Ni—N1—C1	23.86 (15)	C20—C17—C18—C19	176.98 (14)
N4—Ni—N1—C4	−152.39 (9)	C21—C3—C4—N1	178.67 (12)
N4—Ni—N2—C5	81.38 (18)	C21—C3—C4—C5	3.5 (3)
N4—Ni—N2—C9	−88.12 (18)	C21—C22—C23—C24	0.0 (3)
N4—Ni—N3—C10	157.98 (9)	C22—C21—C26—C25	1.7 (2)
N4—Ni—N3—C13	−22.82 (14)	C22—C23—C24—C25	2.0 (2)
C1—N1—C4—C3	−0.98 (15)	C22—C23—C24—C27	−178.30 (17)
C1—N1—C4—C5	175.35 (11)	C23—C24—C25—C26	−2.2 (2)
C1—C2—C3—C4	0.08 (16)	C24—C25—C26—C21	0.4 (2)
C1—C2—C3—C21	−178.02 (13)	C26—C21—C22—C23	−1.9 (2)
C1—C14—C15—C16	176.31 (13)	C27—C24—C25—C26	178.10 (16)
C1—C14—C19—C18	−175.15 (13)	C28—C29—C30—C31	1.4 (2)
C2—C1—C14—C15	−131.52 (15)	C29—C28—C33—C32	−1.42 (19)
C2—C1—C14—C19	44.2 (2)	C29—C30—C31—C32	−1.3 (2)
C2—C3—C4—N1	0.55 (15)	C29—C30—C31—C34	179.26 (13)
C2—C3—C4—C5	−174.66 (15)	C30—C31—C32—C33	−0.3 (2)
C2—C3—C21—C22	73.9 (2)	C31—C32—C33—C28	1.6 (2)
C2—C3—C21—C26	−105.63 (17)	C33—C28—C29—C30	−0.08 (19)
C3—C4—C5—N2	173.97 (14)	C34—C31—C32—C33	179.22 (13)
C3—C4—C5—C6	−5.7 (3)	C35—C11—C12—C13	177.34 (12)
C3—C21—C22—C23	178.53 (14)	C35—C36—C37—C38	0.2 (2)
C3—C21—C26—C25	−178.73 (13)	C36—C35—C40—C39	−0.7 (2)
C4—N1—C1—C2	1.02 (15)	C36—C37—C38—C39	−0.8 (2)
C4—N1—C1—C14	−172.26 (12)	C36—C37—C38—C41	179.97 (14)
C4—C3—C21—C22	−103.77 (17)	C37—C38—C39—C40	0.7 (2)
C4—C3—C21—C26	76.66 (19)	C38—C39—C40—C35	0.1 (2)
C4—C5—C6—C7	−178.31 (13)	C40—C35—C36—C37	0.6 (2)
C5—N2—C9—C8	0.19 (18)	C41—C38—C39—C40	179.91 (14)

### (Acetonitrile-κN){2,2'-(pyridine-2,6-diyl)bis[3,5-bis(4-methylphenyl)pyrrolido-κN]}nickel(II) (pyrr2PyNiCNMe) Crystal data

**Table:**

[Ni(C_41_H_33_N_3_)(C_2_H_3_N)]	*Z* = 2
*M**_r_* = 753.64	*F*(000) = 800
Triclinic, *P*1	*D*_x_ = 1.240 Mg m^−^^3^
*a* = 11.2735 (16) Å	Mo *K*α radiation, λ = 0.71073 Å
*b* = 14.1802 (19) Å	Cell parameters from 8799 reflections
*c* = 14.688 (2) Å	θ = 2.5–32.8°
α = 67.162 (2)°	µ = 0.52 mm^−^^1^
β = 68.881 (2)°	*T* = 100 K
γ = 80.665 (2)°	Prism, colourless
*V* = 2018.0 (5) Å^3^	0.20 × 0.12 × 0.09 mm

### (Acetonitrile-κN){2,2'-(pyridine-2,6-diyl)bis[3,5-bis(4-methylphenyl)pyrrolido-κN]}nickel(II) (pyrr2PyNiCNMe) Data collection

**Table:**

Bruker APEXII CCD diffractometer	7327 reflections with *I* > 2σ(*I*)
Curved-graphite monochromator	*R*_int_ = 0.058
φ and ω scans	θ_max_ = 28.3°, θ_min_ = 2.1°
Absorption correction: multi-scan (SADABS; Bruker, 2016)	*h* = −15→15
*T*_min_ = 0.686, *T*_max_ = 0.899	*k* = −18→18
21869 measured reflections	*l* = −19→19
9994 independent reflections	

### (Acetonitrile-κN){2,2'-(pyridine-2,6-diyl)bis[3,5-bis(4-methylphenyl)pyrrolido-κN]}nickel(II) (pyrr2PyNiCNMe) Refinement

**Table:**

Refinement on *F*^2^	Primary atom site location: dual
Least-squares matrix: full	Secondary atom site location: difference Fourier map
*R*[*F*^2^ > 2σ(*F*^2^)] = 0.068	Hydrogen site location: inferred from neighbouring sites
*wR*(*F*^2^) = 0.193	H-atom parameters constrained
*S* = 1.00	*w* = 1/[σ^2^(*F*_o_^2^) + (0.1214*P*)^2^] where *P* = (*F*_o_^2^ + 2*F*_c_^2^)/3
9994 reflections	(Δ/σ)_max_ = 0.002
551 parameters	Δρ_max_ = 1.75 e Å^−^^3^
178 restraints	Δρ_min_ = −0.93 e Å^−^^3^

### (Acetonitrile-κN){2,2'-(pyridine-2,6-diyl)bis[3,5-bis(4-methylphenyl)pyrrolido-κN]}nickel(II) (pyrr2PyNiCNMe) Special details

**Table:**

Geometry. All esds (except the esd in the dihedral angle between two l.s. planes) are estimated using the full covariance matrix. The cell esds are taken into account individually in the estimation of esds in distances, angles and torsion angles; correlations between esds in cell parameters are only used when they are defined by crystal symmetry. An approximate (isotropic) treatment of cell esds is used for estimating esds involving l.s. planes.

### (Acetonitrile-κN){2,2'-(pyridine-2,6-diyl)bis[3,5-bis(4-methylphenyl)pyrrolido-κN]}nickel(II) (pyrr2PyNiCNMe) Fractional atomic coordinates and isotropic or equivalent isotropic displacement parameters (Å^2^)

**Table:**

	*x*	*y*	*z*	*U*_iso_*/*U*_eq_	Occ. (<1)
Ni	0.71466 (3)	1.00913 (3)	0.03328 (2)	0.02033 (13)	
N1	0.7942 (2)	1.03447 (17)	−0.11157 (17)	0.0212 (5)	
N2	0.6207 (2)	0.91916 (17)	0.02538 (17)	0.0210 (5)	
N3	0.6076 (2)	0.96688 (17)	0.17567 (17)	0.0224 (5)	
N4	0.8317 (2)	1.07621 (18)	0.04797 (16)	0.0229 (5)	
C1	0.9005 (2)	1.0819 (2)	−0.19073 (19)	0.0223 (5)	
C2	0.9285 (3)	1.0467 (2)	−0.2735 (2)	0.0247 (6)	
H2	0.998662	1.066023	−0.336441	0.030*	
C3	0.8342 (3)	0.9777 (2)	−0.2468 (2)	0.0226 (5)	
C4	0.7529 (3)	0.9711 (2)	−0.1464 (2)	0.0222 (5)	
C5	0.9676 (3)	1.1629 (2)	−0.19148 (19)	0.0226 (5)	
C6	0.9035 (3)	1.2478 (2)	−0.1678 (2)	0.0276 (6)	
H6	0.813506	1.253780	−0.150189	0.033*	
C7	0.9693 (3)	1.3232 (2)	−0.1696 (2)	0.0333 (7)	
H7	0.923784	1.379786	−0.152441	0.040*	
C8	1.1014 (3)	1.3173 (2)	−0.1963 (2)	0.0343 (7)	
C9	1.1657 (3)	1.2342 (2)	−0.2225 (2)	0.0311 (7)	
H9	1.255844	1.229402	−0.242326	0.037*	
C10	1.0997 (3)	1.1583 (2)	−0.2200 (2)	0.0266 (6)	
H10	1.145525	1.102123	−0.238019	0.032*	
C11	1.1735 (4)	1.3984 (3)	−0.1962 (3)	0.0535 (10)	
H11A	1.217241	1.441655	−0.268374	0.080*	
H11B	1.236159	1.365374	−0.160591	0.080*	
H11C	1.113791	1.440597	−0.159493	0.080*	
C12	0.8259 (3)	0.9241 (2)	−0.31327 (19)	0.0241 (6)	
C13	0.9345 (3)	0.8812 (2)	−0.3679 (2)	0.0290 (6)	
H13	1.014827	0.888036	−0.364089	0.035*	
C14	0.9268 (3)	0.8288 (2)	−0.4276 (2)	0.0332 (7)	
H14	1.002180	0.800355	−0.464251	0.040*	
C15	0.8110 (3)	0.8167 (2)	−0.4353 (2)	0.0298 (6)	
C16	0.7029 (3)	0.8629 (2)	−0.3836 (2)	0.0296 (6)	
H16	0.622987	0.857461	−0.389079	0.036*	
C17	0.7102 (3)	0.9167 (2)	−0.3242 (2)	0.0273 (6)	
H17	0.635718	0.948668	−0.290877	0.033*	
C18	0.8019 (4)	0.7559 (3)	−0.4967 (3)	0.0419 (8)	
H18A	0.783106	0.684718	−0.450295	0.063*	
H18B	0.882819	0.758244	−0.552870	0.063*	
H18C	0.733704	0.785281	−0.527028	0.063*	
C19	0.6536 (3)	0.9030 (2)	−0.0665 (2)	0.0229 (6)	
C20	0.5975 (3)	0.8240 (2)	−0.0690 (2)	0.0272 (6)	
H20	0.618319	0.811856	−0.132312	0.033*	
C21	0.5111 (3)	0.7634 (2)	0.0217 (2)	0.0296 (6)	
H21	0.471606	0.710148	0.019940	0.035*	
C22	0.4807 (3)	0.7788 (2)	0.1153 (2)	0.0278 (6)	
H22	0.421338	0.736637	0.177326	0.033*	
C23	0.5394 (2)	0.8577 (2)	0.1162 (2)	0.0220 (5)	
C24	0.5332 (3)	0.8847 (2)	0.2030 (2)	0.0222 (5)	
C25	0.4838 (3)	0.8383 (2)	0.3115 (2)	0.0231 (6)	
C26	0.5283 (3)	0.8943 (2)	0.3523 (2)	0.0244 (6)	
H26	0.511937	0.880844	0.424332	0.029*	
C27	0.6013 (3)	0.9739 (2)	0.2682 (2)	0.0223 (5)	
C28	0.3986 (3)	0.7499 (2)	0.3716 (2)	0.0224 (5)	
C29	0.2832 (3)	0.7518 (2)	0.3561 (2)	0.0275 (6)	
H29	0.258054	0.811361	0.307600	0.033*	
C30	0.2044 (3)	0.6683 (2)	0.4102 (2)	0.0280 (6)	
H30	0.126830	0.671260	0.397359	0.034*	
C31	0.2374 (3)	0.5802 (2)	0.4830 (2)	0.0290 (6)	
C32	0.3523 (3)	0.5792 (2)	0.4990 (2)	0.0331 (7)	
H32	0.376789	0.520100	0.548445	0.040*	
C33	0.4316 (3)	0.6622 (2)	0.4446 (2)	0.0290 (6)	
H33	0.509344	0.659241	0.457219	0.035*	
C34	0.1504 (3)	0.4896 (3)	0.5423 (3)	0.0412 (8)	
H34A	0.198295	0.428387	0.572026	0.062*	
H34B	0.116748	0.478387	0.494491	0.062*	
H34C	0.079749	0.503573	0.598715	0.062*	
C35	0.6484 (3)	1.0604 (2)	0.2766 (2)	0.0263 (6)	
C36	0.6888 (3)	1.0435 (3)	0.3615 (2)	0.0336 (7)	
H36	0.689473	0.976019	0.410764	0.040*	
C37	0.7279 (3)	1.1237 (3)	0.3746 (3)	0.0408 (8)	
H37	0.752837	1.110883	0.433680	0.049*	
C38	0.7310 (3)	1.2221 (3)	0.3028 (3)	0.0434 (9)	
C39	0.6875 (3)	1.2401 (3)	0.2198 (3)	0.0410 (8)	
H39	0.686475	1.307769	0.171157	0.049*	
C40	0.6455 (3)	1.1603 (2)	0.2071 (2)	0.0306 (6)	
H40	0.614683	1.174097	0.150804	0.037*	
C41	0.7823 (4)	1.3079 (4)	0.3137 (4)	0.0701 (14)	
H41A	0.772811	1.291198	0.387242	0.105*	
H41B	0.734805	1.371589	0.289428	0.105*	
H41C	0.872489	1.316474	0.271541	0.105*	
C42	0.9054 (3)	1.1128 (2)	0.0596 (2)	0.0229 (6)	
C43	0.9938 (3)	1.1640 (2)	0.0752 (2)	0.0301 (6)	
H43A	0.962221	1.233843	0.069903	0.045*	
H43B	1.077168	1.166375	0.021547	0.045*	
H43C	1.001893	1.126315	0.144527	0.045*	
C1A	0.5344 (10)	0.5285 (8)	−0.2757 (10)	0.090 (3)	0.779 (5)
H1AA	0.557765	0.492990	−0.325469	0.136*	0.779 (5)
H1AB	0.524481	0.602094	−0.312423	0.136*	0.779 (5)
H1AC	0.453946	0.502305	−0.220821	0.136*	0.779 (5)
C1B	0.6389 (7)	0.5100 (5)	−0.2266 (5)	0.0786 (19)	0.779 (5)
H1BA	0.648783	0.435829	−0.188773	0.094*	0.779 (5)
H1BB	0.720963	0.534607	−0.281865	0.094*	0.779 (5)
C1C	0.6032 (7)	0.5668 (6)	−0.1514 (6)	0.0899 (19)	0.779 (5)
H1CA	0.594961	0.640780	−0.190716	0.108*	0.779 (5)
H1CB	0.518935	0.543845	−0.098965	0.108*	0.779 (5)
C1D	0.6971 (6)	0.5521 (5)	−0.0944 (5)	0.0818 (18)	0.779 (5)
H1DA	0.715769	0.477924	−0.064400	0.098*	0.779 (5)
H1DB	0.656402	0.576885	−0.035486	0.098*	0.779 (5)
C1E	0.8225 (6)	0.6066 (5)	−0.1616 (5)	0.0698 (17)	0.779 (5)
H1EA	0.806894	0.679286	−0.200932	0.084*	0.779 (5)
H1EB	0.874385	0.573326	−0.211928	0.084*	0.779 (5)
C1F	0.8895 (11)	0.5976 (8)	−0.0849 (8)	0.114 (3)	0.779 (5)
H1FA	0.976978	0.620915	−0.123713	0.170*	0.779 (5)
H1FB	0.890856	0.526034	−0.038434	0.170*	0.779 (5)
H1FC	0.843881	0.640080	−0.043258	0.170*	0.779 (5)
C1G	0.519 (4)	0.501 (2)	−0.269 (3)	0.063 (5)	0.221 (5)
H1GA	0.508682	0.549644	−0.335107	0.095*	0.221 (5)
H1GB	0.438516	0.466834	−0.223634	0.095*	0.221 (5)
H1GC	0.585205	0.449655	−0.284042	0.095*	0.221 (5)
C1H	0.5578 (19)	0.558 (2)	−0.215 (2)	0.084 (4)	0.221 (5)
H1HA	0.504530	0.534871	−0.140764	0.100*	0.221 (5)
H1HB	0.539431	0.632029	−0.246983	0.100*	0.221 (5)
C1I	0.6978 (19)	0.543 (2)	−0.222 (2)	0.085 (4)	0.221 (5)
H1IA	0.715850	0.469931	−0.187097	0.102*	0.221 (5)
H1IB	0.751851	0.563699	−0.296523	0.102*	0.221 (5)
C1J	0.732 (2)	0.6058 (17)	−0.172 (2)	0.090 (4)	0.221 (5)
H1JA	0.651340	0.636442	−0.137639	0.108*	0.221 (5)
H1JB	0.783165	0.663231	−0.229621	0.108*	0.221 (5)
C1K	0.802 (3)	0.5613 (16)	−0.092 (2)	0.094 (4)	0.221 (5)
H1KA	0.856979	0.501915	−0.103297	0.113*	0.221 (5)
H1KB	0.741166	0.538388	−0.020175	0.113*	0.221 (5)
C1L	0.884 (4)	0.647 (2)	−0.108 (3)	0.078 (5)	0.221 (5)
H1LA	0.941234	0.670951	−0.180777	0.116*	0.221 (5)
H1LB	0.934141	0.620361	−0.060830	0.116*	0.221 (5)
H1LC	0.828534	0.703624	−0.093759	0.116*	0.221 (5)

### (Acetonitrile-κN){2,2'-(pyridine-2,6-diyl)bis[3,5-bis(4-methylphenyl)pyrrolido-κN]}nickel(II) (pyrr2PyNiCNMe) Atomic displacement parameters (Å^2^)

**Table:**

	*U*^11^	*U*^22^	*U*^33^	*U*^12^	*U*^13^	*U*^23^
Ni	0.0217 (2)	0.0251 (2)	0.01235 (18)	−0.00525 (13)	−0.00082 (13)	−0.00728 (14)
N1	0.0227 (11)	0.0236 (11)	0.0153 (10)	−0.0031 (9)	−0.0035 (9)	−0.0066 (9)
N2	0.0209 (11)	0.0276 (12)	0.0133 (10)	−0.0047 (9)	−0.0018 (8)	−0.0079 (9)
N3	0.0225 (11)	0.0271 (12)	0.0163 (10)	−0.0034 (9)	−0.0016 (9)	−0.0095 (9)
N4	0.0243 (11)	0.0282 (12)	0.0111 (10)	−0.0064 (9)	0.0010 (8)	−0.0058 (9)
C1	0.0210 (13)	0.0272 (14)	0.0125 (11)	−0.0021 (11)	−0.0009 (10)	−0.0043 (10)
C2	0.0250 (13)	0.0289 (14)	0.0151 (12)	−0.0008 (11)	−0.0017 (10)	−0.0070 (11)
C3	0.0247 (13)	0.0273 (14)	0.0135 (12)	0.0010 (11)	−0.0042 (10)	−0.0075 (10)
C4	0.0250 (13)	0.0261 (14)	0.0148 (12)	−0.0011 (11)	−0.0054 (10)	−0.0075 (10)
C5	0.0245 (13)	0.0260 (13)	0.0115 (11)	−0.0038 (11)	−0.0018 (10)	−0.0030 (10)
C6	0.0301 (15)	0.0299 (15)	0.0190 (13)	−0.0029 (12)	−0.0058 (11)	−0.0060 (11)
C7	0.0455 (18)	0.0264 (15)	0.0245 (15)	−0.0033 (13)	−0.0066 (13)	−0.0090 (12)
C8	0.0450 (18)	0.0346 (16)	0.0188 (14)	−0.0156 (14)	−0.0057 (13)	−0.0036 (12)
C9	0.0283 (15)	0.0400 (17)	0.0195 (13)	−0.0083 (13)	−0.0047 (11)	−0.0050 (12)
C10	0.0281 (14)	0.0294 (15)	0.0156 (12)	−0.0033 (11)	−0.0024 (11)	−0.0047 (11)
C11	0.065 (3)	0.053 (2)	0.045 (2)	−0.028 (2)	−0.0103 (19)	−0.0168 (18)
C12	0.0298 (14)	0.0270 (14)	0.0106 (11)	−0.0032 (11)	−0.0002 (10)	−0.0066 (10)
C13	0.0274 (14)	0.0364 (16)	0.0198 (13)	−0.0018 (12)	−0.0016 (11)	−0.0119 (12)
C14	0.0363 (17)	0.0378 (17)	0.0204 (14)	0.0005 (13)	0.0010 (12)	−0.0153 (13)
C15	0.0395 (17)	0.0325 (15)	0.0142 (12)	−0.0067 (13)	−0.0008 (11)	−0.0100 (11)
C16	0.0325 (15)	0.0398 (16)	0.0179 (13)	−0.0049 (13)	−0.0063 (11)	−0.0120 (12)
C17	0.0304 (15)	0.0353 (15)	0.0142 (12)	0.0002 (12)	−0.0030 (11)	−0.0111 (11)
C18	0.055 (2)	0.0460 (19)	0.0255 (16)	−0.0127 (16)	−0.0008 (15)	−0.0210 (15)
C19	0.0243 (13)	0.0290 (14)	0.0132 (12)	0.0002 (11)	−0.0030 (10)	−0.0085 (10)
C20	0.0299 (15)	0.0353 (15)	0.0169 (13)	−0.0067 (12)	−0.0039 (11)	−0.0110 (12)
C21	0.0316 (15)	0.0347 (16)	0.0232 (14)	−0.0116 (12)	−0.0042 (12)	−0.0114 (12)
C22	0.0270 (14)	0.0328 (15)	0.0189 (13)	−0.0110 (12)	0.0000 (11)	−0.0073 (12)
C23	0.0208 (13)	0.0268 (13)	0.0137 (12)	−0.0027 (10)	−0.0004 (10)	−0.0065 (10)
C24	0.0217 (13)	0.0260 (13)	0.0165 (12)	−0.0038 (10)	−0.0019 (10)	−0.0079 (10)
C25	0.0204 (13)	0.0289 (14)	0.0165 (12)	−0.0021 (11)	−0.0014 (10)	−0.0081 (11)
C26	0.0238 (13)	0.0330 (15)	0.0148 (12)	−0.0027 (11)	−0.0016 (10)	−0.0105 (11)
C27	0.0222 (13)	0.0282 (14)	0.0146 (12)	−0.0020 (11)	−0.0024 (10)	−0.0085 (10)
C28	0.0241 (13)	0.0243 (13)	0.0144 (12)	−0.0039 (11)	0.0001 (10)	−0.0069 (10)
C29	0.0290 (14)	0.0274 (14)	0.0186 (13)	−0.0016 (11)	−0.0043 (11)	−0.0033 (11)
C30	0.0250 (14)	0.0346 (15)	0.0223 (14)	−0.0048 (12)	−0.0035 (11)	−0.0104 (12)
C31	0.0340 (16)	0.0301 (15)	0.0202 (13)	−0.0075 (12)	−0.0019 (12)	−0.0101 (12)
C32	0.0402 (17)	0.0275 (15)	0.0252 (15)	−0.0031 (13)	−0.0100 (13)	−0.0024 (12)
C33	0.0275 (14)	0.0333 (15)	0.0241 (14)	−0.0036 (12)	−0.0077 (12)	−0.0074 (12)
C34	0.045 (2)	0.0354 (17)	0.0371 (18)	−0.0169 (15)	−0.0078 (15)	−0.0053 (14)
C35	0.0221 (13)	0.0374 (16)	0.0189 (13)	−0.0037 (12)	0.0020 (10)	−0.0164 (12)
C36	0.0269 (15)	0.054 (2)	0.0222 (14)	−0.0069 (14)	0.0008 (11)	−0.0220 (14)
C37	0.0291 (16)	0.066 (2)	0.0367 (18)	−0.0086 (15)	−0.0001 (13)	−0.0357 (18)
C38	0.0357 (18)	0.055 (2)	0.047 (2)	−0.0112 (15)	0.0054 (15)	−0.0389 (18)
C39	0.0355 (17)	0.0375 (18)	0.046 (2)	−0.0045 (14)	0.0027 (15)	−0.0235 (16)
C40	0.0254 (14)	0.0332 (16)	0.0290 (15)	−0.0041 (12)	0.0008 (12)	−0.0145 (13)
C41	0.064 (3)	0.081 (3)	0.084 (3)	−0.019 (2)	0.000 (2)	−0.064 (3)
C42	0.0268 (14)	0.0238 (13)	0.0143 (12)	−0.0032 (11)	−0.0034 (10)	−0.0049 (10)
C43	0.0299 (15)	0.0332 (16)	0.0292 (15)	−0.0073 (12)	−0.0112 (12)	−0.0095 (13)
C1A	0.094 (6)	0.093 (6)	0.098 (5)	−0.016 (5)	−0.010 (5)	−0.061 (5)
C1B	0.092 (4)	0.070 (4)	0.068 (3)	−0.007 (3)	−0.014 (3)	−0.027 (3)
C1C	0.104 (4)	0.080 (4)	0.082 (4)	−0.008 (3)	−0.023 (3)	−0.030 (3)
C1D	0.087 (4)	0.070 (3)	0.082 (4)	−0.006 (3)	−0.020 (3)	−0.027 (3)
C1E	0.078 (4)	0.059 (3)	0.063 (3)	−0.012 (3)	−0.010 (3)	−0.020 (3)
C1F	0.121 (6)	0.121 (7)	0.107 (7)	−0.036 (7)	−0.012 (6)	−0.060 (6)
C1G	0.084 (10)	0.062 (10)	0.076 (9)	−0.002 (9)	−0.048 (8)	−0.038 (8)
C1H	0.098 (6)	0.085 (6)	0.076 (6)	−0.003 (6)	−0.026 (6)	−0.038 (5)
C1I	0.095 (5)	0.080 (5)	0.077 (5)	−0.008 (5)	−0.023 (5)	−0.028 (5)
C1J	0.094 (5)	0.081 (5)	0.079 (5)	−0.006 (5)	−0.017 (5)	−0.020 (5)
C1K	0.097 (6)	0.084 (6)	0.087 (6)	−0.008 (6)	−0.017 (6)	−0.025 (6)
C1L	0.097 (10)	0.072 (11)	0.078 (10)	−0.033 (10)	−0.028 (9)	−0.031 (9)

### (Acetonitrile-κN){2,2'-(pyridine-2,6-diyl)bis[3,5-bis(4-methylphenyl)pyrrolido-κN]}nickel(II) (pyrr2PyNiCNMe) Geometric parameters (Å, º)

**Table:**

Ni—N1	1.896 (2)	C30—C31	1.394 (4)
Ni—N2	1.846 (2)	C31—C32	1.394 (4)
Ni—N3	1.906 (2)	C31—C34	1.515 (4)
Ni—N4	1.861 (2)	C32—H32	0.9500
N1—C1	1.375 (3)	C32—C33	1.386 (4)
N1—C4	1.397 (3)	C33—H33	0.9500
N2—C19	1.365 (3)	C34—H34A	0.9800
N2—C23	1.362 (3)	C34—H34B	0.9800
N3—C24	1.390 (3)	C34—H34C	0.9800
N3—C27	1.376 (3)	C35—C36	1.401 (4)
N4—C42	1.140 (3)	C35—C40	1.391 (4)
C1—C2	1.404 (4)	C36—H36	0.9500
C1—C5	1.469 (4)	C36—C37	1.385 (4)
C2—H2	0.9500	C37—H37	0.9500
C2—C3	1.407 (4)	C37—C38	1.381 (5)
C3—C4	1.403 (4)	C38—C39	1.393 (5)
C3—C12	1.485 (4)	C38—C41	1.512 (5)
C4—C19	1.445 (4)	C39—H39	0.9500
C5—C6	1.398 (4)	C39—C40	1.393 (4)
C5—C10	1.392 (4)	C40—H40	0.9500
C6—H6	0.9500	C41—H41A	0.9800
C6—C7	1.384 (4)	C41—H41B	0.9800
C7—H7	0.9500	C41—H41C	0.9800
C7—C8	1.394 (5)	C42—C43	1.449 (4)
C8—C9	1.391 (5)	C43—H43A	0.9800
C8—C11	1.512 (4)	C43—H43B	0.9800
C9—H9	0.9500	C43—H43C	0.9800
C9—C10	1.386 (4)	C1A—H1AA	0.9800
C10—H10	0.9500	C1A—H1AB	0.9800
C11—H11A	0.9800	C1A—H1AC	0.9800
C11—H11B	0.9800	C1A—C1B	1.532 (6)
C11—H11C	0.9800	C1B—H1BA	0.9900
C12—C13	1.394 (4)	C1B—H1BB	0.9900
C12—C17	1.395 (4)	C1B—C1C	1.515 (5)
C13—H13	0.9500	C1C—H1CA	0.9900
C13—C14	1.382 (4)	C1C—H1CB	0.9900
C14—H14	0.9500	C1C—C1D	1.515 (5)
C14—C15	1.393 (4)	C1D—H1DA	0.9900
C15—C16	1.397 (4)	C1D—H1DB	0.9900
C15—C18	1.506 (4)	C1D—C1E	1.525 (5)
C16—H16	0.9500	C1E—H1EA	0.9900
C16—C17	1.393 (4)	C1E—H1EB	0.9900
C17—H17	0.9500	C1E—C1F	1.526 (5)
C18—H18A	0.9800	C1F—H1FA	0.9800
C18—H18B	0.9800	C1F—H1FB	0.9800
C18—H18C	0.9800	C1F—H1FC	0.9800
C19—C20	1.391 (4)	C1G—H1GA	0.9800
C20—H20	0.9500	C1G—H1GB	0.9800
C20—C21	1.382 (4)	C1G—H1GC	0.9800
C21—H21	0.9500	C1G—C1H	1.529 (6)
C21—C22	1.387 (4)	C1H—H1HA	0.9900
C22—H22	0.9500	C1H—H1HB	0.9900
C22—C23	1.395 (4)	C1H—C1I	1.530 (6)
C23—C24	1.443 (4)	C1I—H1IA	0.9900
C24—C25	1.396 (4)	C1I—H1IB	0.9900
C25—C26	1.399 (4)	C1I—C1J	1.519 (6)
C25—C28	1.481 (4)	C1J—H1JA	0.9900
C26—H26	0.9500	C1J—H1JB	0.9900
C26—C27	1.401 (4)	C1J—C1K	1.526 (6)
C27—C35	1.476 (4)	C1K—H1KA	0.9900
C28—C29	1.393 (4)	C1K—H1KB	0.9900
C28—C33	1.392 (4)	C1K—C1L	1.533 (6)
C29—H29	0.9500	C1L—H1LA	0.9800
C29—C30	1.387 (4)	C1L—H1LB	0.9800
C30—H30	0.9500	C1L—H1LC	0.9800
			
N1—Ni—N3	166.68 (9)	C31—C32—H32	119.2
N2—Ni—N1	83.38 (9)	C33—C32—C31	121.6 (3)
N2—Ni—N3	83.52 (9)	C33—C32—H32	119.2
N2—Ni—N4	168.06 (10)	C28—C33—H33	119.7
N4—Ni—N1	96.77 (9)	C32—C33—C28	120.6 (3)
N4—Ni—N3	96.54 (10)	C32—C33—H33	119.7
C1—N1—Ni	138.26 (19)	C31—C34—H34A	109.5
C1—N1—C4	106.5 (2)	C31—C34—H34B	109.5
C4—N1—Ni	113.43 (17)	C31—C34—H34C	109.5
C19—N2—Ni	118.35 (18)	H34A—C34—H34B	109.5
C23—N2—Ni	117.87 (18)	H34A—C34—H34C	109.5
C23—N2—C19	122.5 (2)	H34B—C34—H34C	109.5
C24—N3—Ni	112.48 (17)	C36—C35—C27	119.3 (3)
C27—N3—Ni	138.85 (19)	C40—C35—C27	122.2 (3)
C27—N3—C24	106.1 (2)	C40—C35—C36	118.3 (3)
C42—N4—Ni	176.7 (2)	C35—C36—H36	119.6
N1—C1—C2	109.5 (2)	C37—C36—C35	120.9 (3)
N1—C1—C5	124.8 (2)	C37—C36—H36	119.6
C2—C1—C5	125.5 (2)	C36—C37—H37	119.6
C1—C2—H2	126.0	C38—C37—C36	120.8 (3)
C1—C2—C3	107.9 (2)	C38—C37—H37	119.6
C3—C2—H2	126.0	C37—C38—C39	118.6 (3)
C2—C3—C12	125.8 (2)	C37—C38—C41	120.4 (4)
C4—C3—C2	105.8 (2)	C39—C38—C41	120.9 (4)
C4—C3—C12	128.4 (3)	C38—C39—H39	119.5
N1—C4—C3	110.2 (2)	C38—C39—C40	121.0 (3)
N1—C4—C19	113.8 (2)	C40—C39—H39	119.5
C3—C4—C19	134.9 (3)	C35—C40—C39	120.3 (3)
C6—C5—C1	122.1 (2)	C35—C40—H40	119.9
C10—C5—C1	119.9 (3)	C39—C40—H40	119.9
C10—C5—C6	117.9 (3)	C38—C41—H41A	109.5
C5—C6—H6	119.6	C38—C41—H41B	109.5
C7—C6—C5	120.8 (3)	C38—C41—H41C	109.5
C7—C6—H6	119.6	H41A—C41—H41B	109.5
C6—C7—H7	119.5	H41A—C41—H41C	109.5
C6—C7—C8	121.1 (3)	H41B—C41—H41C	109.5
C8—C7—H7	119.5	N4—C42—C43	176.7 (3)
C7—C8—C11	121.2 (3)	C42—C43—H43A	109.5
C9—C8—C7	118.2 (3)	C42—C43—H43B	109.5
C9—C8—C11	120.6 (3)	C42—C43—H43C	109.5
C8—C9—H9	119.6	H43A—C43—H43B	109.5
C10—C9—C8	120.8 (3)	H43A—C43—H43C	109.5
C10—C9—H9	119.6	H43B—C43—H43C	109.5
C5—C10—H10	119.4	H1AA—C1A—H1AB	109.5
C9—C10—C5	121.2 (3)	H1AA—C1A—H1AC	109.5
C9—C10—H10	119.4	H1AB—C1A—H1AC	109.5
C8—C11—H11A	109.5	C1B—C1A—H1AA	109.5
C8—C11—H11B	109.5	C1B—C1A—H1AB	109.5
C8—C11—H11C	109.5	C1B—C1A—H1AC	109.5
H11A—C11—H11B	109.5	C1A—C1B—H1BA	109.9
H11A—C11—H11C	109.5	C1A—C1B—H1BB	109.9
H11B—C11—H11C	109.5	H1BA—C1B—H1BB	108.3
C13—C12—C3	120.6 (3)	C1C—C1B—C1A	109.1 (7)
C13—C12—C17	118.2 (3)	C1C—C1B—H1BA	109.9
C17—C12—C3	121.2 (3)	C1C—C1B—H1BB	109.9
C12—C13—H13	119.6	C1B—C1C—H1CA	108.6
C14—C13—C12	120.8 (3)	C1B—C1C—H1CB	108.6
C14—C13—H13	119.6	H1CA—C1C—H1CB	107.6
C13—C14—H14	119.2	C1D—C1C—C1B	114.6 (6)
C13—C14—C15	121.5 (3)	C1D—C1C—H1CA	108.6
C15—C14—H14	119.2	C1D—C1C—H1CB	108.6
C14—C15—C16	117.6 (3)	C1C—C1D—H1DA	108.5
C14—C15—C18	121.5 (3)	C1C—C1D—H1DB	108.5
C16—C15—C18	120.9 (3)	C1C—C1D—C1E	114.9 (6)
C15—C16—H16	119.4	H1DA—C1D—H1DB	107.5
C17—C16—C15	121.1 (3)	C1E—C1D—H1DA	108.5
C17—C16—H16	119.4	C1E—C1D—H1DB	108.5
C12—C17—H17	119.7	C1D—C1E—H1EA	110.7
C16—C17—C12	120.6 (3)	C1D—C1E—H1EB	110.7
C16—C17—H17	119.7	C1D—C1E—C1F	105.3 (6)
C15—C18—H18A	109.5	H1EA—C1E—H1EB	108.8
C15—C18—H18B	109.5	C1F—C1E—H1EA	110.7
C15—C18—H18C	109.5	C1F—C1E—H1EB	110.7
H18A—C18—H18B	109.5	C1E—C1F—H1FA	109.5
H18A—C18—H18C	109.5	C1E—C1F—H1FB	109.5
H18B—C18—H18C	109.5	C1E—C1F—H1FC	109.5
N2—C19—C4	110.6 (2)	H1FA—C1F—H1FB	109.5
N2—C19—C20	119.0 (2)	H1FA—C1F—H1FC	109.5
C20—C19—C4	130.2 (2)	H1FB—C1F—H1FC	109.5
C19—C20—H20	120.4	H1GA—C1G—H1GB	109.5
C21—C20—C19	119.1 (3)	H1GA—C1G—H1GC	109.5
C21—C20—H20	120.4	H1GB—C1G—H1GC	109.5
C20—C21—H21	119.3	C1H—C1G—H1GA	109.5
C20—C21—C22	121.4 (3)	C1H—C1G—H1GB	109.5
C22—C21—H21	119.3	C1H—C1G—H1GC	109.5
C21—C22—H22	120.8	C1G—C1H—H1HA	108.6
C21—C22—C23	118.4 (2)	C1G—C1H—H1HB	108.6
C23—C22—H22	120.8	C1G—C1H—C1I	114.6 (17)
N2—C23—C22	119.5 (2)	H1HA—C1H—H1HB	107.6
N2—C23—C24	110.7 (2)	C1I—C1H—H1HA	108.6
C22—C23—C24	129.7 (2)	C1I—C1H—H1HB	108.6
N3—C24—C23	114.4 (2)	C1H—C1I—H1IA	109.2
N3—C24—C25	110.6 (2)	C1H—C1I—H1IB	109.2
C25—C24—C23	134.3 (3)	H1IA—C1I—H1IB	107.9
C24—C25—C26	105.9 (2)	C1J—C1I—C1H	112.2 (15)
C24—C25—C28	127.3 (3)	C1J—C1I—H1IA	109.2
C26—C25—C28	126.8 (2)	C1J—C1I—H1IB	109.2
C25—C26—H26	126.1	C1I—C1J—H1JA	106.4
C25—C26—C27	107.9 (2)	C1I—C1J—H1JB	106.4
C27—C26—H26	126.1	C1I—C1J—C1K	123.6 (18)
N3—C27—C26	109.5 (2)	H1JA—C1J—H1JB	106.5
N3—C27—C35	124.7 (2)	C1K—C1J—H1JA	106.4
C26—C27—C35	125.3 (2)	C1K—C1J—H1JB	106.4
C29—C28—C25	120.9 (2)	C1J—C1K—H1KA	110.4
C33—C28—C25	121.1 (3)	C1J—C1K—H1KB	110.4
C33—C28—C29	118.0 (2)	C1J—C1K—C1L	106.8 (15)
C28—C29—H29	119.4	H1KA—C1K—H1KB	108.6
C30—C29—C28	121.3 (3)	C1L—C1K—H1KA	110.4
C30—C29—H29	119.4	C1L—C1K—H1KB	110.4
C29—C30—H30	119.5	C1K—C1L—H1LA	109.5
C29—C30—C31	120.9 (3)	C1K—C1L—H1LB	109.5
C31—C30—H30	119.5	C1K—C1L—H1LC	109.5
C30—C31—C34	120.6 (3)	H1LA—C1L—H1LB	109.5
C32—C31—C30	117.6 (3)	H1LA—C1L—H1LC	109.5
C32—C31—C34	121.8 (3)	H1LB—C1L—H1LC	109.5
			
Ni—N1—C1—C2	161.2 (2)	C10—C5—C6—C7	−1.9 (4)
Ni—N1—C1—C5	−23.5 (4)	C11—C8—C9—C10	178.2 (3)
Ni—N1—C4—C3	−166.90 (18)	C12—C3—C4—N1	−179.7 (3)
Ni—N1—C4—C19	3.1 (3)	C12—C3—C4—C19	13.3 (5)
Ni—N2—C19—C4	−6.0 (3)	C12—C13—C14—C15	−0.1 (5)
Ni—N2—C19—C20	169.9 (2)	C13—C12—C17—C16	3.5 (4)
Ni—N2—C23—C22	−170.8 (2)	C13—C14—C15—C16	2.4 (4)
Ni—N2—C23—C24	6.5 (3)	C13—C14—C15—C18	−177.4 (3)
Ni—N3—C24—C23	−7.9 (3)	C14—C15—C16—C17	−1.7 (4)
Ni—N3—C24—C25	163.61 (19)	C15—C16—C17—C12	−1.3 (4)
Ni—N3—C27—C26	−156.8 (2)	C17—C12—C13—C14	−2.8 (4)
Ni—N3—C27—C35	31.3 (5)	C18—C15—C16—C17	178.1 (3)
N1—Ni—N2—C19	6.3 (2)	C19—N2—C23—C22	−4.1 (4)
N1—Ni—N2—C23	173.5 (2)	C19—N2—C23—C24	173.1 (2)
N1—C1—C2—C3	2.0 (3)	C19—C20—C21—C22	−1.0 (5)
N1—C1—C5—C6	−48.1 (4)	C20—C21—C22—C23	0.2 (5)
N1—C1—C5—C10	134.1 (3)	C21—C22—C23—N2	2.3 (4)
N1—C4—C19—N2	1.7 (3)	C21—C22—C23—C24	−174.3 (3)
N1—C4—C19—C20	−173.7 (3)	C22—C23—C24—N3	178.1 (3)
N2—Ni—N1—C1	−167.0 (3)	C22—C23—C24—C25	9.2 (5)
N2—Ni—N1—C4	−4.96 (19)	C23—N2—C19—C4	−172.6 (2)
N2—C19—C20—C21	−0.7 (4)	C23—N2—C19—C20	3.3 (4)
N2—C23—C24—N3	1.3 (3)	C23—C24—C25—C26	169.7 (3)
N2—C23—C24—C25	−167.6 (3)	C23—C24—C25—C28	−11.9 (5)
N3—Ni—N1—C1	−177.4 (4)	C24—N3—C27—C26	2.5 (3)
N3—Ni—N1—C4	−15.3 (5)	C24—N3—C27—C35	−169.4 (3)
N3—Ni—N2—C19	−176.1 (2)	C24—C25—C26—C27	1.1 (3)
N3—Ni—N2—C23	−8.8 (2)	C24—C25—C28—C29	−55.6 (4)
N3—C24—C25—C26	0.5 (3)	C24—C25—C28—C33	123.9 (3)
N3—C24—C25—C28	178.9 (3)	C25—C26—C27—N3	−2.2 (3)
N3—C27—C35—C36	−152.3 (3)	C25—C26—C27—C35	169.6 (3)
N3—C27—C35—C40	32.6 (4)	C25—C28—C29—C30	178.4 (3)
N4—Ni—N1—C1	1.0 (3)	C25—C28—C33—C32	−178.8 (3)
N4—Ni—N1—C4	163.01 (19)	C26—C25—C28—C29	122.5 (3)
N4—Ni—N2—C19	−85.1 (5)	C26—C25—C28—C33	−58.0 (4)
N4—Ni—N2—C23	82.1 (5)	C26—C27—C35—C36	37.1 (4)
C1—N1—C4—C3	0.7 (3)	C26—C27—C35—C40	−138.0 (3)
C1—N1—C4—C19	170.7 (2)	C27—N3—C24—C23	−173.4 (2)
C1—C2—C3—C4	−1.5 (3)	C27—N3—C24—C25	−1.8 (3)
C1—C2—C3—C12	178.7 (3)	C27—C35—C36—C37	−176.9 (3)
C1—C5—C6—C7	−179.7 (3)	C27—C35—C40—C39	178.1 (3)
C1—C5—C10—C9	179.3 (2)	C28—C25—C26—C27	−177.3 (3)
C2—C1—C5—C6	126.5 (3)	C28—C29—C30—C31	0.9 (4)
C2—C1—C5—C10	−51.2 (4)	C29—C28—C33—C32	0.7 (4)
C2—C3—C4—N1	0.5 (3)	C29—C30—C31—C32	−0.2 (4)
C2—C3—C4—C19	−166.5 (3)	C29—C30—C31—C34	179.5 (3)
C2—C3—C12—C13	43.0 (4)	C30—C31—C32—C33	−0.3 (5)
C2—C3—C12—C17	−135.9 (3)	C31—C32—C33—C28	0.0 (5)
C3—C4—C19—N2	168.3 (3)	C33—C28—C29—C30	−1.1 (4)
C3—C4—C19—C20	−7.0 (5)	C34—C31—C32—C33	−180.0 (3)
C3—C12—C13—C14	178.2 (3)	C35—C36—C37—C38	−1.6 (5)
C3—C12—C17—C16	−177.5 (3)	C36—C35—C40—C39	2.9 (4)
C4—N1—C1—C2	−1.7 (3)	C36—C37—C38—C39	3.4 (5)
C4—N1—C1—C5	173.7 (2)	C36—C37—C38—C41	−175.8 (3)
C4—C3—C12—C13	−136.8 (3)	C37—C38—C39—C40	−2.0 (5)
C4—C3—C12—C17	44.3 (4)	C38—C39—C40—C35	−1.1 (5)
C4—C19—C20—C21	174.3 (3)	C40—C35—C36—C37	−1.6 (4)
C5—C1—C2—C3	−173.3 (3)	C41—C38—C39—C40	177.1 (3)
C5—C6—C7—C8	0.8 (4)	C1A—C1B—C1C—C1D	178.1 (7)
C6—C5—C10—C9	1.5 (4)	C1B—C1C—C1D—C1E	72.3 (8)
C6—C7—C8—C9	0.8 (4)	C1C—C1D—C1E—C1F	169.8 (7)
C6—C7—C8—C11	−178.7 (3)	C1G—C1H—C1I—C1J	177 (2)
C7—C8—C9—C10	−1.3 (4)	C1H—C1I—C1J—C1K	130 (3)
C8—C9—C10—C5	0.1 (4)	C1I—C1J—C1K—C1L	148 (3)
